# A dual-model SERS and RRS analytical platform for Pb(II) based on Ag-doped carbon dot catalytic amplification and aptamer regulation

**DOI:** 10.1038/s41598-019-46426-y

**Published:** 2019-07-10

**Authors:** Haidong Wang, Xiaowei Huang, Guiqing Wen, Zhiliang Jiang

**Affiliations:** Key Laboratory of Ecology of Rare and Endangered Species and Environmental Protection (Guangxi Normal University), Ministry of Education, Guangxi Key Laboratory of Environmental Pollution Control Theory and Technology, Guilin, 541004 China

**Keywords:** Environmental chemistry, Environmental impact

## Abstract

Several carbon dots doping with diferent elements (Ca, Ag, Au) were fabricated and their catalytic properties had been investigated in this paper. It was found that the Ag-doped carbon dots (CD_Ag_) had played a role of mimic enzyme on the reaction of HAuCl_4_-H_2_O_2_ and generated nanogold particles with surface enhanced Raman scattering (SERS) and resonance Rayleigh scattering (RRS) effects. The aptamer (Apt) can be adsorbed on the CD_Ag_ surface and cause the catalysis weakening. When the target Pb(II) was added, it would combine with the Apt to produce firm complexes Pb-Apt and desorb CD_Ag_, which caused its catalytic effect restore. The formed nanogold had a strong RRS peak (at 375 nm) and a high SERS peak (at 1615 cm^−1^) in the presence of molecular probe (Victoria blue B, VBB). The dual-model signals of SERS and RRS increased linearly with Pb(II) concentration increase within the scope of 0.006–0.46 μmol/L and 0.01–0.46 μmol/L. And their detection limits respectively were 0.0032 μmol/L and 0.0048 μmol/L Pb(II).

## Introduction

Aptamers are a class of oligonucleotides with single-strand that can be binded specifically with a variety of target molecules^1,2^. It has the characteristics of convenient modification, easy obtainment, toilless storing. And it has attracted more research focus in analysis because it can specifically combine with many kinds of targets such as proteins, metal ions and organic substances^3–5^. Qian *et al*.^6^ modified the Pb(II) aptamer on the reduced graphene quantum dots and established a new fluorescence method to detect 9.9–435 nmol/L Pb(II). In the report of Ye *et al*.^7^, the double-stranded DNA was unfolded by lead to produce a single nucleic acid aptamer, which wrapped on the surface of nanogold. So that nanogold can be stably dispersed in the sodium chloride solution. As a result, the system resonance scatter value decreased and 16.7–666.7 nmol/L lead ion was quantitfied. Chen *et al*.^8^ have reported an absorption spectral method to determine amphetamine by aptamer-heme system. In a polar organic solvent, heme has a strong absorption peak, which can be combined with random DNA by π-π and deposited into nanoparticles. Upon addition of target heme, aggregation occurs and the absorbance of the system was reduced. Based on this, 0.5–40 μg/L heme could be detected with the detection limit (DL) of 0.2 μg/L. Combining with a three-dimensional (3D) DNA walker, Lv *et al*.^9^ have designed a new photoelectrochemical (PEC) method based on ‘Z-scheme’ systems to ultrasensitively detect prostate-specific antigen (PSA). The analysis system had sensitive photocurrent answers to 0.01−50 ng/mL PSA, and the DL was low to 1.5 pg/mL. With inducing conformation conversion through DNA hybridization, “Z-scheme” PEC biosensor was used by Zeng *et al*.^10^ to establish a novel and well designed palindromic molecular beacon (PMB) to selectively screen kanamycin (Kana). The above mesurement system gave sensitive photocurrent responses with the Kana concentration change in the range of 50–5000 fM, and the DL was 29 fM.

SERS refers to a phenomenon of Raman scattering signal enhancement caused by the adsorption of certain molecules on the surface of nano-sized rough metal^11–13^. Combining the high selectivity of the aptamer reaction with the highly sensitive SERS is a major innovation in nanoplasma, which has achieved good results. Gao *et al*.^14^ reported a new label-free SERS method to detect bacteria. The aptamer was used as a template for the *in situ* preparation of nanosiver particles by aptamers interacting with their specific binding bacteria. SERS signal can be enhanced by aptamer binding bacteria to achieve the purpose of detecting bacteria. The detection range was 10 to 10^7^ cfu/mL, and the DL was up to 1.5 cfu/mL. Wen *et al*.^15^ can measure 6.3–403.6 μg/L melamine using the Ag-aptamer complex as a SERS probe. Ouyang *et al*.^16^ reported a aptamer coupled SERS tenique for the determination of 0.13–53.33 nmol/L Pb(II) by using the catalysis of gold nanoparticle (AuNP) on the reduction of HAuCl_4_-H_2_O_2_. Du *et al*.^17^ covered specific aptamers of gonadotropin progesterone (P4) on nanogold surface and lead to the dispersion of AuNPs in NaCl solution. When P4 was present, it can be combined with an aptamer to form a stable complex structure, which releases AuNPs to aggregate in solution. A 2.6–1400 nmol/L P4 can be detected by colorimetry. Dual-model method is more stable and sensitive than the single model methods and has attracted more and more attentions^18–20^. For example, Zhang *et al*.^18^ demonstrated the determination of alkaline phosphatase activity (APA) with a dual-mode (colorimetric and fluorescent) method by sensing with a kind of composite core-shell nanoparticles. This dual-mode study provided a new sensing tenique with the well application of the optical properties of AuNPs and the luminescence properties of polymers. Combining hybridization chain reaction (HCR) with multifunctional AuNPs, Yu *et al*.^19^ presented an ultrasensitive colorimetric/fluorescent sensor to detect Hg^2+^, and the detetion limits of the two modes were both below 10 nM. RRS, a convenient and sensitive molecular spectroscopy, can combine with fluorescence to develop some sensitive fluorescence and RRS dual-model methods^21,22^. For example, based on the changes of fluorescence and RRS intensity after the addition of albendazole (ABZ) to CdTe quantum dots system, Li *et al*. proposed a dual-model fluorescence/RRS technique to determine ABZ with the DL of nanogram level^21^. Fu *et al*. prepared a handle substrate of cellulose paper for telomerase with using a dual-mode method of colorimetry and upconversion fluorescence^23^. Based on the linear change of RRS intensity with the addition of Bi(III) since the reduction of Bi(III) will consume I_3_^−^ and there was energy transfer (RRS-ET) between I_3_^−^ and graphene oxide (GO), Liang *et al*. built a dual-model RRS/SERS method for Bi(III) with a linear range of 0.05–5.5 μmol/L and a DL of 4 ng/mL^24^. However, the SERS and RRS dual-model for trace Pb(II) that based on the catalytic effect of CD_Ag_ on H_2_O_2_ reduction of HAuCl_4_ has rarely been reported.

The heavy metal Lead is an important pollutant, which has many sources of pollution and exists in water, atmosphere or biota. It can harm the human hematopoietic system, nervous system and kidneys^25–27^, causing various diseases. Lead can cause poisoning when the concentration of lead in the blood exceeds 480 nmol/L. Therefore, it is great significance for detecting Pb^2+^ in environmental protection and human health. Now, there are several methods for detecting Pb^2+^ such as atomic absorption spectrometry (AAS), fluorescence spectroscopy (FL), inductively coupled plasma mass spectrometry (ICP-MS), and spectrophotometry, etc.^28–32^. However, the application of these technologies is limited due to low sensitivity or the need for expensive instruments. So, it remains a challenge for the determination of trace lead ion (II) to design a new method. Chai *et al*.^33^ reported a simple, cheap and sensitive colorimetric method that can detect 100 nmol/L Pb^2+^. Niu *et al*.^34^ reported an “on” fluorescence sensor for detecting Pb^2+^ using the energy transfer between AuNP and graphene quantum dots. The linear range of Pb^2+^ was 50–4000 nmol/L with a DL of 16.7 nmol/L. Yu *et al*.^35^ reported a deoxyribonuclease cleavage effect: Pb-Pt alloy-functionalized Fe-MOFs were used to amplify Pb^2+^. The liner range was 0.005–1000 nmol/L and the DL was up to 2 pmol/L. In this paper, a rapid and sensitive SERS method for detection of Pb^2+^ was established by aptamer-regulated CD_Ag_-catalyzed H_2_O_2_ oxidation of HAuCl_4_. Zhou *et al*.^36^ built a very sensitive electrochemical biosensor for Pb^2+^ by using the catalysis of DNAzyme on the reduction of H_2_O_2_. To increase the sensitivity, thionine was used as the electron mediator and porors Au-Pd nanoparticles were introduced to enhance the detected signal^37^. As a result, the current variation (ΔI) of the cathodic peak linearly changed with Pb^2+^ concentration in the range of 1.0–100 nM and the DL was 0.34 pM. Wu *et al*.^38^ developed an original electrochemiluminescence (ECL) method to detect the activity of *Escherichia coli* formamidopyrimidine-DNA glycosylase (FPG). Based on the linear change of ECL intensity with FPG concentration, a range of 0–4.0 U/mL target could be detected. Combining DNA-based HCR with a specific DNAzyme, Zhuang *et al*.^39^ established a new electrochemical DNA biosensor based on magnetic control to ultrasensitively detect lead. The new method could detect 0.1–75 nM Pb^2+^, and the DL was 37 pM.

## Results

### SERS spectra

**Figure 1:**
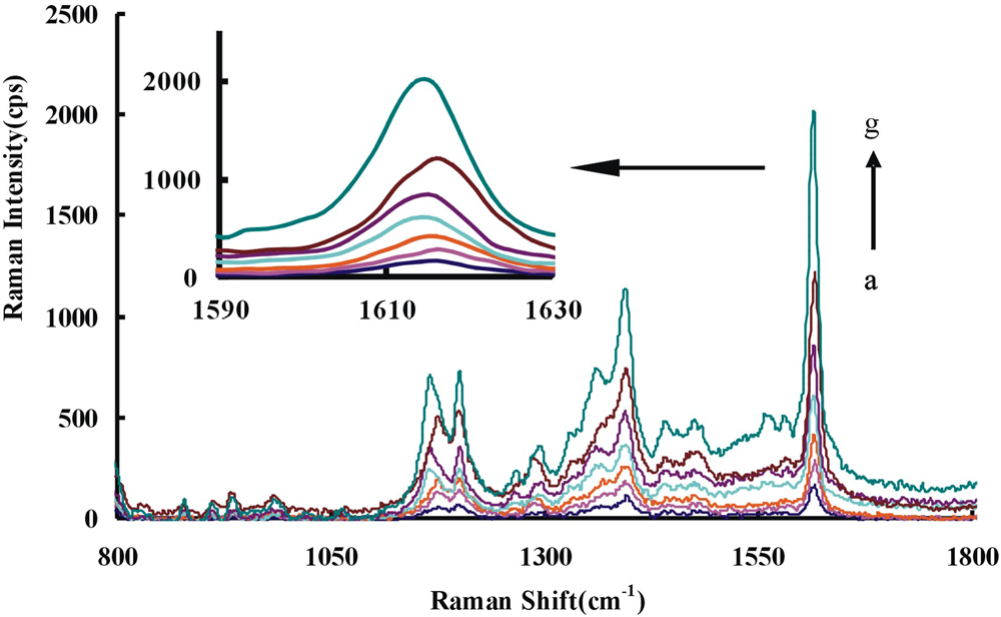
SERS spectra of Apt-Pb(II)-CD_Ag2_-HAuCl_4_-H_2_O_2_-HCl-VBB system (**a**) 80 nmol/L Apt + 0.36 mg/mL CD_Ag2_ + 0.16 mmol/L HCl + 11.2 μmol/L HAuCl_4_ + 2 mmol/L H_2_O_2_ + 0.27 μmol/L VBB; (**b**) a + 0.006 μmol/L Pb(II); (**c**) a + 0.03 μmol/L Pb(II); (**d**) a + 0.06 μmol/L Pb(II); (**e**) a + 0.13 μmol/L Pb(II); (**f**) a + 0.23 μmol/L Pb(II); (**g**) a + 0.46 μmol/L Pb(II).

The reduction of HAuCl_4_ by H_2_O_2_ is slow. The CD_Ca_/CD _Ag0-3_/CD_Au0-4_ can strongly catalyze the redox reaction between H_2_O_2_ and HAuCl_4_. The results showed that the more CD added the more Au nanoparticle produced. The five mainly SERS peaks of the above system with VBB as molecular probe were at 1176 cm^−1^, 1198 cm^−1^,1365 cm^−1^, 1391 cm^−1^ and 1615 cm^−1^ (Figs S1–S11). The two peaks of 1176 cm^−1^ and 1198 cm^−1^ could be attributed to the C-N bond’s stretching vibration and telescopic vibration respectively^40^. While the two peaks of 1365 cm^−1^ and 1391 cm^−1^ could be due to the stretching vibration of C-H. The most repeatable and sensitive peak (at 1615 cm^−1^), which was caused by the vibration of C=C of the benzene ring, was selected. The CD_Ag1-3_-HAuCl_4_-H_2_O_2_-VBB system did not have a SERS signal without heating (Fig. S4). So, it proves that it is not a SERS signal generated by CD_Ag_. In the aptamer-regulated system, the aptamer can specifically bind to the catalyst, inhibiting its catalytic action and linearly reducing the SERS signals of the system (Fig. S12). After adding Pb(II), it can bind to Apt, which activates the catalytic activity of CD_Ca_/CD_Ag0-3_/CD_Au2_ and accelerates the reaction. The number of produced Au nanoparticles increased with Pb ions concentration increase. The SERS activity enhanced linearly with Pb(II) concentration increase when VBB was used as probe molecule (Figs 1, S13–S17). In the experiment, AgNO_3_ was involved to prepare CD_Ag_ and glucose was used as the carbon source. Ag^+^ is attracted to the surface of the CD by electrostatic interaction with the functional groups. The d orbital of Ag interacts with the p/π orbital of C to form a stable CD. The doping of Ag can change the surface electrons of CD and make the electron transfer between reactants easier. Thereby their catalytic activity increase and facilitate the reaction of H_2_O_2_-HAuCl_4_. By comparing the tested CDs, CD_Ag2_ had the strongest catalytic effect and its detection system was most sensitive (Fig. 1).

### Resonance Rayleigh scattering (RRS) spectra and absorption(Abs) spectra

**Figure 2:**
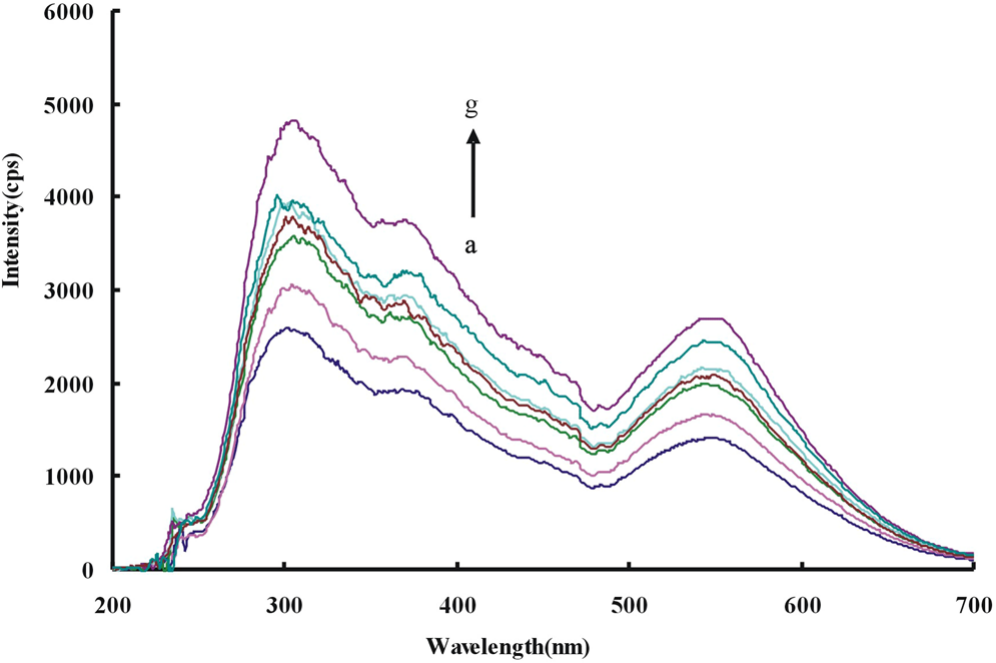
RRS spectra of Apt-Pb(II)-CD_Ag2_-HAuCl_4_-H_2_O_2_-HCl system (**a**) 80 nmol/L Apt + 0.36 mg/mL CD_Ag2_ + 0.16 mmol/L HCl + 11.2 μmol/L HAuCl_4_ + 2 mmol/L H_2_O_2_; (**b**) a + 0.01 μmol/L Pb(II); (**c**) a + 0.06 μmol/L Pb(II); (**d**) a + 0.15 μmol/L Pb(II); (**e**) a + 0.2 μmol/L Pb(II); (**f**) a + 0.33 μmol/L Pb(II); (**g**) a + 0.46 μmol/L Pb(II).

To get RRS spectra, the reaction systems were synchronously scanned with a fluorescence spectrophotometer. For the Apt-Pb(II)-CD_Ca_/CD_Ag_-HAuCl_4_-H_2_O_2_ system, there were 3 strong peaks at 305 nm, 375 nm and 540 nm. Among them, the peaks of 375 nm and 540 nm are characteristic peaks of Au nanoparticles while the peak of 305 nm was generated by the light source of instrument. So the characteristic peak (at 375 nm) with higher peak strength was selected to investigate. Due to the specifical binding of Pb(II) with Apt, more Pb(II) adding lead to more CD release. More Au nanoparticles were generated and the RRS signal was gradually enhanced (Fig. 2). Because the CDs can catalyze the reduction of HAuCl_4_ by H_2_O_2_, the formed Au nanoparticles increased with the CDs increase, and so did the RRS signal (Fig. S18). The Apt inhibition on the CDs catalysis was discussed too, and the results showed that the Apt inhibited the catalytic action of CDs and linearly reducing the RRS activity of the system (Fig. S19).

**Figure 3:**
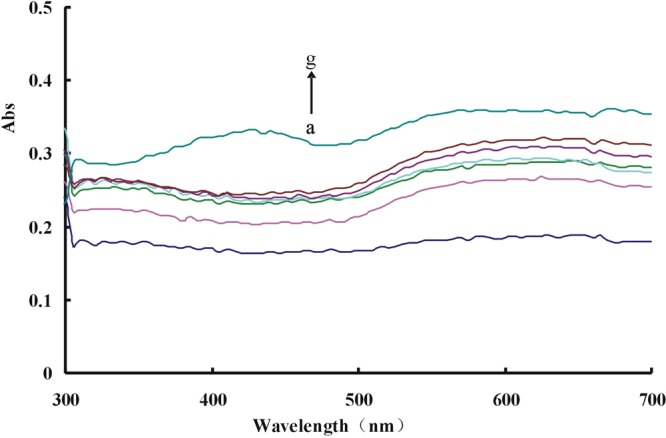
Abs spectra of Apt-Pb(II)-CD_Ag2_-HAuCl_4_-H_2_O_2_-HCl system (**a**) 80 nmol/L Apt + 0.36 mg/mL CD_Ag2_ + 0.16 mmol/L HCl + 11.2 μmol/L HAuCl_4_ + 2 mmol/L H_2_O_2_; (**b**) a + 0.01 μmol/L Pb(II); (**c**) a + 0.06 μmol/L Pb(II); (**d**) a + 0.1 μmol/L Pb(II); (**e**) a + 0.13 μmol/L Pb(II); (**f**) a + 0.16 μmol/L Pb(II); (**g**) a + 0.20 μmol/L Pb(II).

The Abs spectra were obtained with the chosen conditions. For the Apt- Pb(II)-CD_Ag_-HAuCl_4_ -H_2_O_2_ system, there was a wide peak at about 575 nm, which gradually enhanced with Pb(II) concentration increase (Fig. 3). The binding of CD_Ca_/CD_Ag_ with Apt inhibited the catalysis in the absence of Pb(II) (Fig. S20). The CDs catalysis was recorded by Abs spectra at the same time, and the absorption gradually increased with the concentration of CD_Ca_/CD_Ag_ increases (Fig. S21).

### Spectral characteristics and stability of CD_Ag2_

The CD_Ag2_, which had the strongest catalytic activity aomong the above CDs, was selected as the representative for the experiments of spectral characteristics and stability. The prepared CDs was allowed to stand at room temperature for several days, which were measured at the maximum Abs, RRS, fluorescence (Flu) and SERS values at different times. Results indicates that the prepared CDs were stable, which relative standard deviation (RSD) was within 10% for 12 days (Table 1).

**Table 1:** Stability of CD_Ag2_.

Signal	1 day	2 day	4 day	8 day	12 day	Averange	RSD (%)
A_422_	0.164	0.159	0.175	0.169	0.172	0.168	3.8
I_450 nm_	670.3	662.3	689.2	669.6	670.2	672.3	1.5
F_442nm_	725.8	700.6	736.8	735.9	725.6	724.9	2.0
I_1615cm-1_	758.2	780.5	790.5	776.2	800.3	781.1	2.0

To investigate whether the SERS signal was produced by Au nanoparticles, a product of the redox reaction, or by the aggregated CDs, the stability test of CDs at NaCl solution was performed. The results showed that only high concentrations of CD_Ag2_ will aggregate in the NaCl solution (Table 2).The SERS signal I_Ag2/Au_ at 1615cm^−1^ rises to a gentle in different concentration gradient NaCl solution. The SERS signal of high concentration CD_Au2_ in NaCl solution was weak and there was no obvious aggregation phenomenon. Therefore, it can be proved that the SERS signals in the experimental system came from Au nanoparticles, but not CDs.

**Table 2:** Stability of CD_Ag2_ and CD_Au_ in NaCl solution*.

C_NaCl_ (mol/L)	0	0.01	0.05	0.1	0.5	Averange	RSD (%)
I _Ag2-1615cm-1_	360	558	769	872	603	632.4	31.3
I_/Au-1615cm-1_	72	77	73	79	68	73.8	5.86

*CD_Ag2_:3.33 mg/mL; CD_Au3_:4.8 mg/mL.

**Figure 4:**
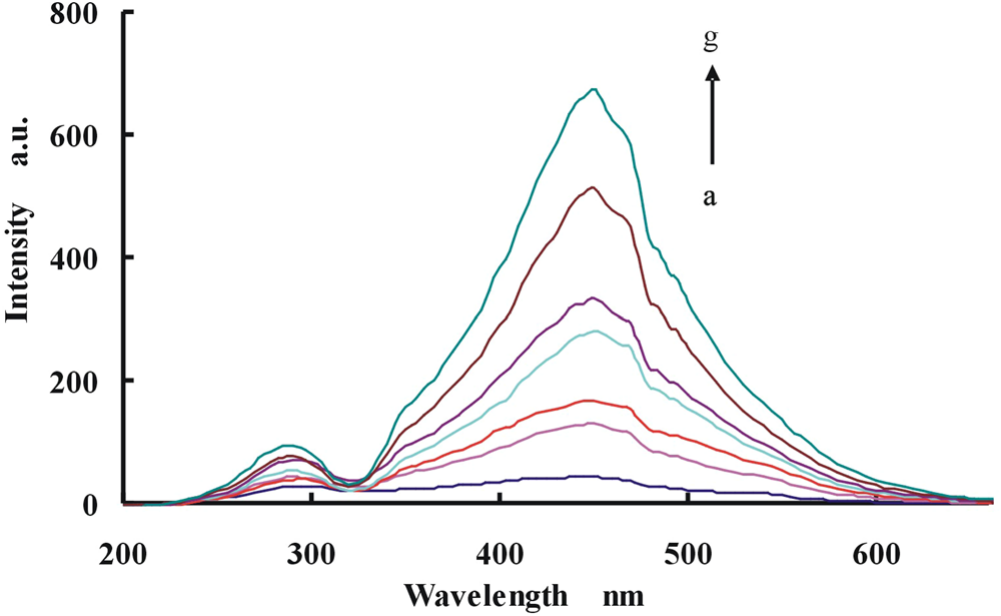
RRS spectra of CD_Ag2_ (**a**) 0 mg/mL CD_Ag2_; (**b**) 0.165 mg/mL CD_Ag2_; (**c**) 0.33 mg/mL CD_Ag2_; (**d**) 0.5 mg/mL CD_Ag2_; (**e**) 0.66 mg/mL CD_Ag2_; (**f**) 1.0 mg/mL CD_Ag2_; (**g**)1.25 mg/mL CD_Ag2_.

**Figure 5:**
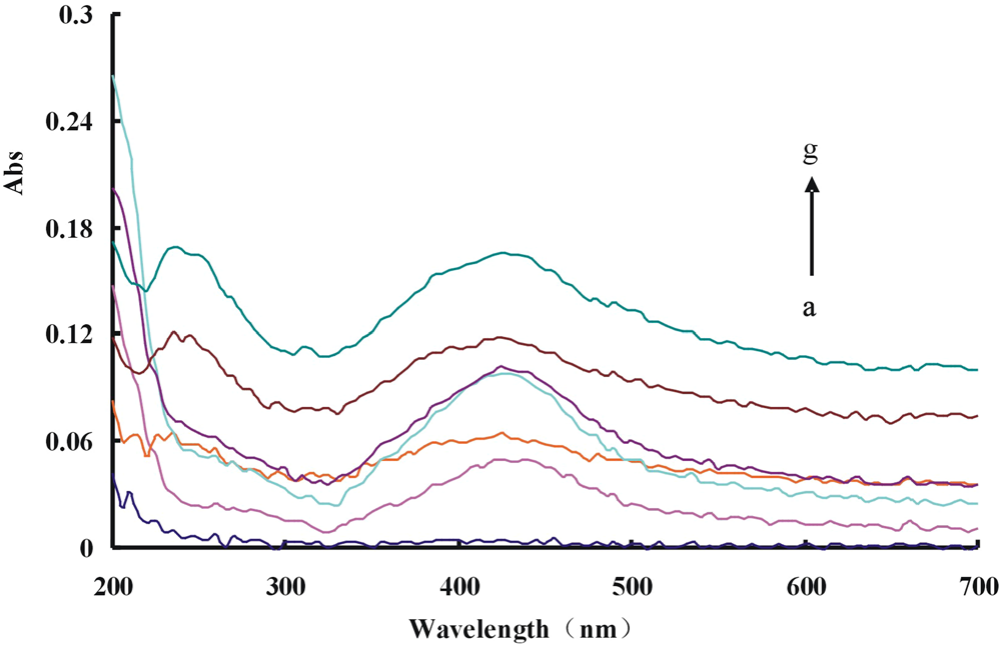
Absorption spectra of CD_Ag2_ (**a**) 0 mg/mL CD_Ag2_; (**b**) 0.165 mg/mL CD_Ag2_; (**c**) 0.33 mg/mL CD_Ag2_; (**d**) 0.5 mg/mL CD_Ag2_; (**e**) 0.66 mg/mL CD_Ag2_; (**f**) 1.0 mg/mL CD_Ag2_; (**g**) 1.25 mg/mL CD_Ag2_.

**Figure 6:**
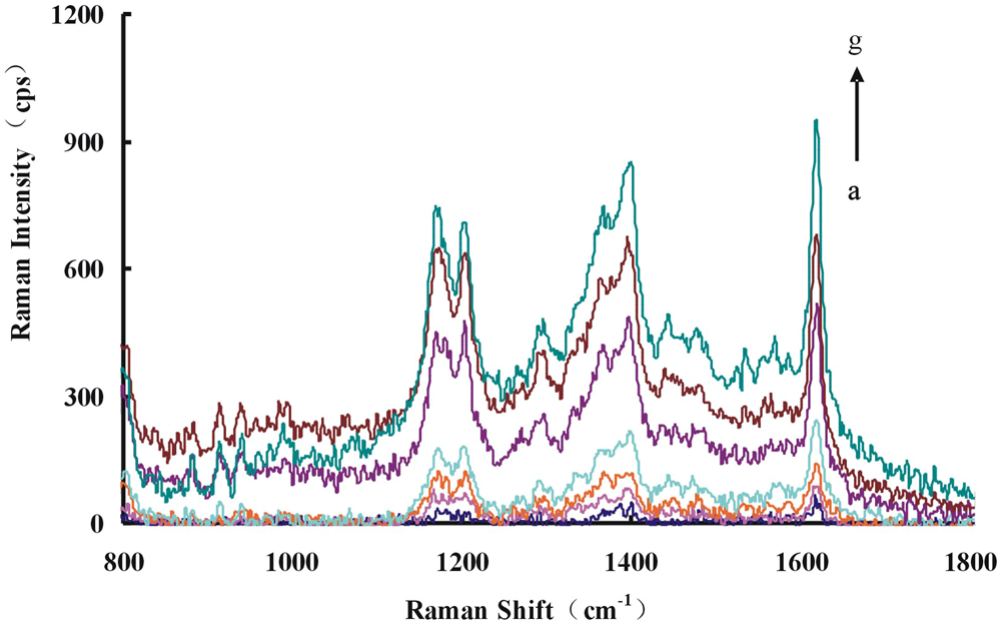
SERS spectra of CD_Ag2_ (**a**) 0.27μmol/L VBB; (**b**) a + 1.65 mg/mL CD_Ag2_; (**c**) a + 3.33 mg/mL CD_Ag2_; (**d**) a + 5.25 mg/mL CD_Ag2_; (**e**) a + 6.67 mg/mL CD_Ag2_; (**f**) a + 10 mg/mL CD_Ag2_; (**g**) a + 12.5 mg/mL CD_Ag2_.

The RRS spectra of CD_Ag2_ were measured. The characteristic RRS peak of CD_Ag2_ was at 450 nm while there was another peak at 295 nm (Fig. 4). The intensities of the characteristic peak (at 450 nm) gradually increase as the concentration of CD_Ag2_ increased. The Abs spectra of CD_Ag2_ were investigated and its characteristic peak (at 425 nm) increased with CD_Ag2_ concentration increase (Fig. 5). From the SERS spectra of CD_Ag2_ (Fig. 6), we can see that the characteristic peak of CD_Ag2_ was at 1615 cm^−1^, which the peak intensity increases linearly with the CD_Ag2_ concentration increase.

### CDs catalysis and aptamer inhibition

The CD_Ca_/CD_Ag0-3_/CD_Au0-4_ catalytical effects on the redox reaction of HAuCl_4_-H_2_O_2_ were studied (Table S1). The values of the SERS/RRS/UV peak had a positive correlation with the catalyst concentration. The CD_Ag2_-HAuCl_4_ -H_2_O_2_ system has the strongest catalytic ability. When Apt was added to the system to inhibit its catalysis, the SERS/RRS/UV signal weakened. The system ΔI_1615cm-1_/ΔI_375nm_/ΔA_575nm_ had linear relation with the aptamer concentration (Table S2).

### Scanning electron microscopy (SEM) and laser scattering

**Figure 7:**
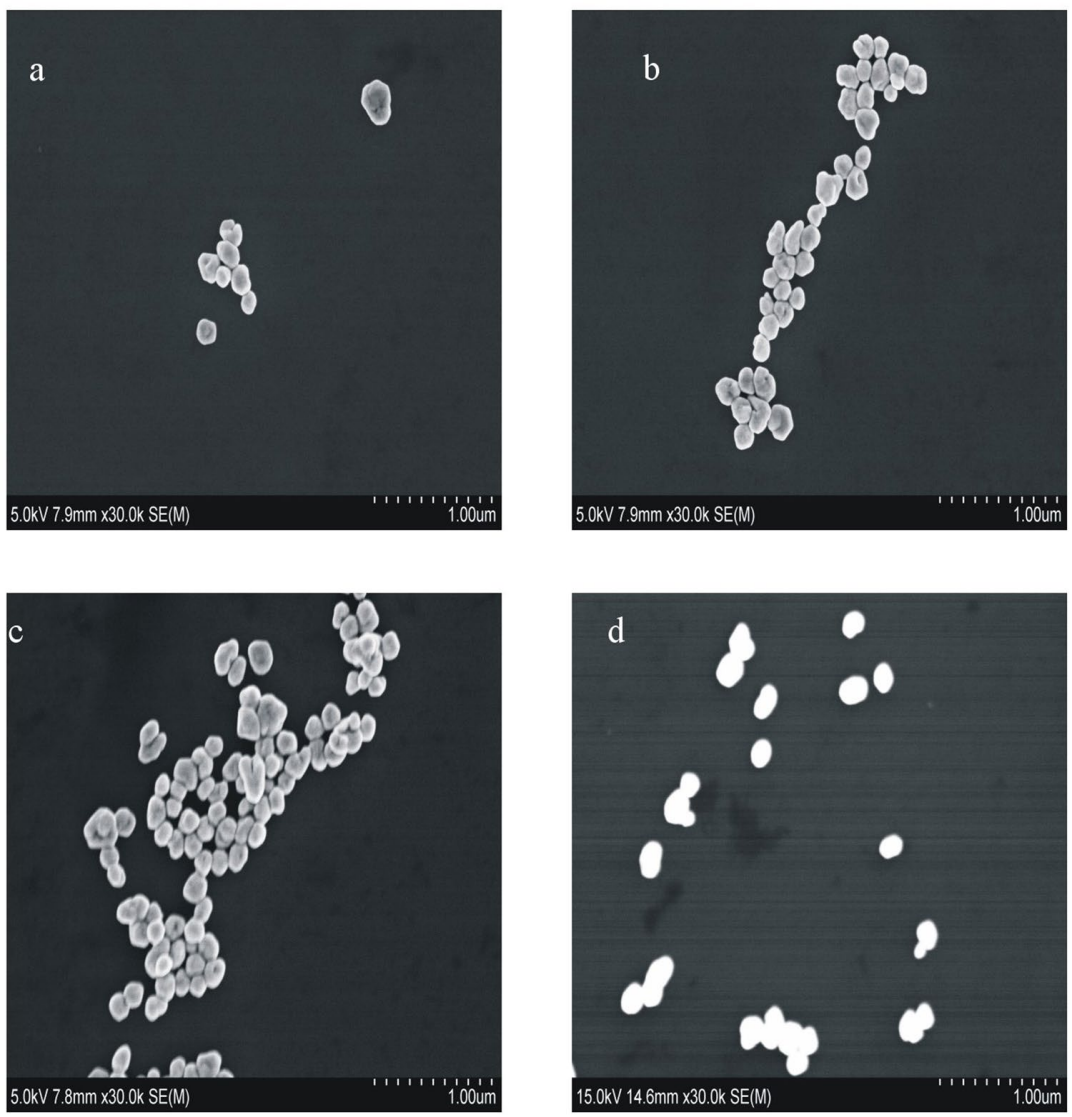
SEM of the reaction system and CD_Ag2_ (**a**) 80 nmol/L Apt + 0.36 mg/mL CD_Ag2_ + 0.16 mmol/L HCl + 11.2 μmol/L HAuCl_4_ + 2 mmol/L H_2_O_2_; (**b**) a + 0.13 μmol/L Pb(II); (**c**) a + 0.46 μmol/L Pb(II); (**d**) 0.36 mg/mL CD_Ag2_.

According to the experimental method, SEM and laser scattering of the concerned samples were tested. For the Apt-Pb(II)-CD_Ag2_-HAuCl_4_-H_2_O_2_-HCl system, the Apt had inhibits on CD_Ag2_ catalysis in the absence of Pb(II). So the catalytic effect on the reaction of H_2_O_2_ - HAuCl_4_ was less, and so did the product of nanogold. In this case, the particles was smaller and their average size was about 90 nm (Fig. 7a). With the increase of Pb(II) concentration, CD_Ag2_ was gradually released, and the produced nanogold particles increased. The average sizes of two systems with different Pb(II) concentration grew up to about 140 nm and 160 nm respectively (Fig. 7b,c). A SEM result showed that the average particle size of CD_Ag2_ was near 40 nm (Fig. 7d).

**Figure 8:**
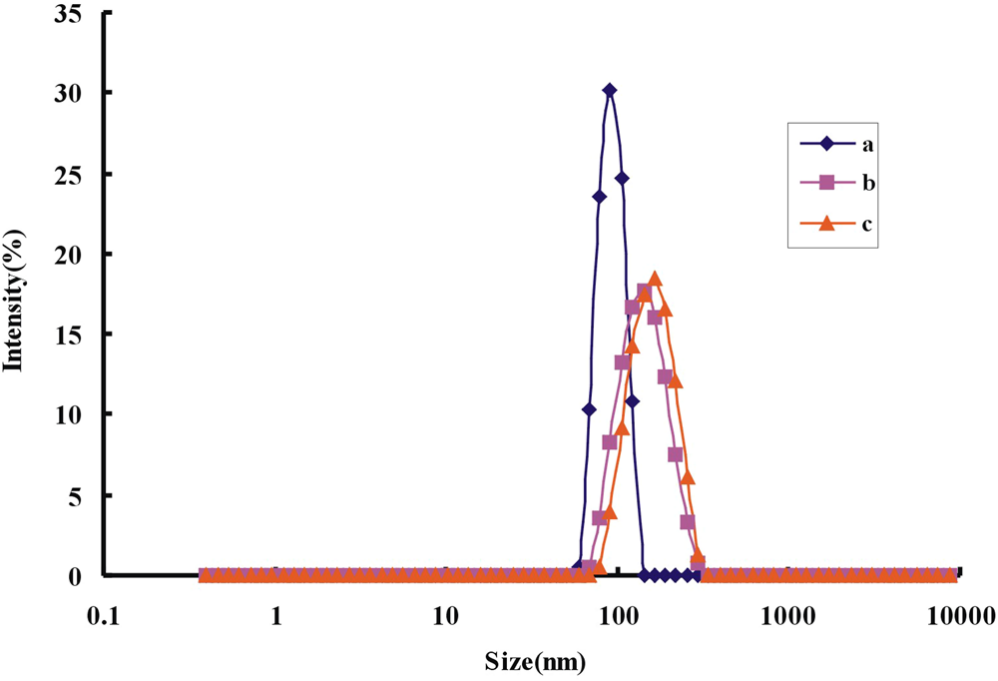
Particle size distribution of nanoparticles (**a**) 80 nmol/L Apt + 0.36 mg/mL CD_Ag2_ + 0.16 mmol/L HCl + 11.2 μmol/L HAuCl_4_ + 2 mmol/L H_2_O_2_; (**b**) a + 0.13 μmol/L Pb(II); (**c**) a + 0.46 μmol/L Pb(II).

Laser scattering was used to quickly record the size distribution of the tested samples. In our work, the particle size, size distribution and potential distribution of nanoparticles were accurately measured. The results of particle size experiments by laser scattering were consistent with that of SEM (Fig. 8). The surface charge distribution experiments showed that the charge distribution of the anlasis system was −1.31 mV when there was no Pb(II). As the Pb(II) concentration increases, the resulting nanoparticle charge distribution was −12 mV (Fig. S22). In the analysis system, CD_Ag2_ was gradually released with Pb(II) concentration increase, and the aggregate of Au nanoparticles was enhanced. The charge distribution was gradually increased with Pb(II) concentration increase, and the system tends to be stable.

### Optimization of analysis condition

The analysis conditions of the Apt-Pb(II)-CD_Ag2_-HAuCl_4_-H_2_O_2_-VBB system were optimized. The influences of reactants concentration on SERS signal (Δ*I*_1615cm-1_) were tested (Figs S23–S27). And acorrding to the results, the optimal concentrations of 0.36 mg/mL CD_Ag2_, 67 nmol/L Apt, 0.16 mmol/L HCl, 11.2 μmol/L HAuCl_4_, and 2 mmol/L H_2_O_2_ were used. Besides, the effects of reaction conditions were optimized, and a 15 min reaction time and a 60 °C reaction temperature were chosen in the system (Figs S28, S29). What’s more, a 0.27 μmol/L VBB was used (Fig. S30).

### Effect of coexistent substance

The effects of coexisting substances (CS) of the Apt-Pb(II)-CD_Ag2_-HAuCl_4_-H_2_O_2_-HCl-VBB system on the detection of 0.1 μM Pb^2+^ were investigated. The results showed that 1000 times ([CS]/[Pb]) K^+^, Ca^2+^, Zn^2+^, Cl^−^, Cu^2+^, NH_4_^+^, 500 times Na^+^, Cr^3+^, SO_4_^2−^, S_2_O_3_^2−^, CO_3_^2−^, PO_4_^3−^, 100 times Ba^2+^, Mg^2+^, NO_2_^−^, Co^2+^, Hg^2+^, 50 times Al^3+^, Cr^6+^ and 10 times Fe^2+^ did not affect the measurement when the relative error was in the scope of −10–10% (Table S3). So, this method was very selective.

### Working curve

Under the optimal conditions, the working curves of different SERS/RRS/Abs systems were plotted according to experimental methods (Table 3). In the SERS systems, working curves of between Pt(II) concentration and their corresponding ΔI were plotted for six systems of Apt-CD_Ca_/CD_Ag0-3_/CD_Au2_-HAuCl_4_-H_2_O_2_-HCl-VBB (Fig. S31). The results show that the slope of the Apt- Pb(II)-CD_Ag2_-HAuCl_4_-H_2_O_2_- HCl system is the largest, so the most sensitive system can be used to determine 0.006–0.46 μmol/L Pb(II) by SERS method. Its linear equation is ΔI_1615cm-1_ = 3858 C + 113.01, with the correlation coefficient of 0.9865 and the DL of 0.0032 μmol/L Pb. In the RRS method (Fig. S32), the results show that the slope of the Apt-CD_Ag2_-HAuCl_4_-H_2_O_2_- HCl system is the largest, so the most sensitive system can be used to detect 0.01–0.46 μmol/L Pb. The linear equation is ΔI = 3458.2 C + 222.66, with the correlation coefficient of 0.9602 and the DL of 0.0048 μmol/L Pb. In the Abs detection method (Fig. S33), the slope of the Apt-CD_Ag2_-HAuCl_4_-H_2_O_2_- HCl system is the largest, so the most sensitive system can be used to mesure 0.01–0.46 μmol/L Pb by Abs method. The linear equation is ΔA_575nm_ = 0.8032 C + 0.0127, with the coefficient of 0.9562, and the DL of 0.0048 μmol/L. Among all the above techniques of SERS/RRS/UV, the SERS method has the highest sensitivity, and the Abs method is the most convenient and cheap. This paper chooses SERS method to measure Pb(II). By comparing the reported molecular spectrometric methods of Pb(II), the SERS method of this paper has the advantage of simple, rapid, and high sensitivity (Table S4)^41–45^.

**Table 3:** Analysis characteristics of SERS and RRS detection of Pb(II).

System	Method	Linear range μmol//L	Regress equation	Coefficient	DL (μmol//L)
Apt-CD_Ca_	SERS	0.02–0.46	ΔI_1615cm-1_ = 1170.3 C + 35.0	0.9793	0.018
	RRS	0.033–0.67	ΔI_375nm_ = 2418.6 C + 77.4	0.9707	0.017
Apt-CD_Ag0_	SERS	0.013–0.46	ΔI_1614cm-1_ = 2620.4 C + 43.0	0.976	0.007
	RRS	0.026–0.6	ΔI_375nm_ = 481.24 + 4.1	0.9825	0.013
Apt-CD_Ag1_	SERS	0.013–0.4	ΔI_1614cm-1_ = 2991.2 C + 67.5	0.9872	0.068
	RRS	0.026–0.46	ΔI_375nm_ = 2974.6 C + 278.7	0.9349	0.012
Apt-CD_Ag2_	SERS	0.006–0.46	ΔI_1615cm-1_ = 3858 C + 113.0	0.9865	0.0032
	RRS	0.01–0.46	ΔI_375nm_ = 3458.2 C + 222.7	0.9602	0.0048
Apt-CD_Ag3_	SERS	0.026–0.53	ΔI_1615cm-1_ = 2225 C + 5.0	0.9809	0.014
	RRS	0.033–0.6	ΔI_375nm_ = 1162.2 C + 73.8	0.9754	0.016
Apt-CD_Au2_	SERS	0.026–0.53	ΔI_1619cm-1_ = 1758.1 C + 40.9	0.9417	0.012

*DL: detection limit.

### Analysis of samples

Take 100 mL of mineral water, tap water, wastewater of Bokanglou Laboratory of Guangxi Normal University and the pond water of Yanshan Campus of Guangxi Normal University, respectively, to determine Pb content according to the procedures. The results showed that the recovery was 94.6–100.7%, with a relative standard deviation of 2.10–4.92% (Table 4). The national drinking water standard stipulates that Pb^2+^ ≤ 0.01 mg/L, and the maximum allowable emission concentration in wastewater is Pb^2+^ ≤ 0.05 mg/L. So, it indicates that the Pb^2+^ contents in the samples were in line with national standards.

**Table 4:** Analysis of Pb(II) Samples.

Sample	Content (μmol/L)	Added (μmol/L)	Found (μmol/L, n = 5)	Average (μmol/L)	RSD (%)	Recovery (%)
Mineral water	—	0.067	0.059, 0.063, 0.071, 0.065, 0.060	0.064	3.81	95.5
Tap water	—	0.111	0.107, 0.109, 0.103, 0.099, 0.107	0.105	4.92	94.6
Pond water	—	0.133	0.132, 0.129, 0.135, 0.134, 0.138	0.134	2.52	100.7
Wast water	0.052	0.133	0.181, 0.188, 0.179, 0.184, 0.179	0.182	2.10	98.4

## Discussion

### Analysis principle

**Figure 9:**
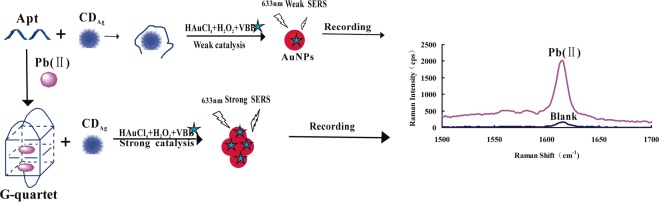
Principle of aptamer regulated SERS detection of Pb(II) based on CD_Ag_ catalysis.

The CDs can strongly catalyze the redox reaction of H_2_O_2_-HAuCl_4_. While the Apt can envelop the CDs to weaken its catalytic action. The target molecule Pb(II) can specifically bind with the Apt, which lead to the release of the CDs and the recovery of their catalytic activity. There was a proportional relationship between SERS intensity and Pb(II) concentration with using VBB as molecular probes. Accordingly, an original SERS technique for determination of Pb(II) was created (Fig. 9).

## Conclusions

In an acidic environment, the reduction of HAuCl_4_ by H_2_O_2_ is slow. Under the catalysis of CD_Ag2_, the reaction occurs rapidly and gold nanoparticles are formed. After the addition of the probe VBB to the system, there is a strong SERS signal. When Pb(II) is added, A very stable complex was formed between Pb(II) and the aptamer, which lead to linearly enhance of absorbance, RRS and SERS intensity. Based on this, an aptamer regulated- SERS and RRS dual-model method for Pb(II) by exploiting CD catalyzing the reduction of HAuCl_4_-H_2_O_2_ was established. The analysis system was highly sensitive, highly selective, simple and rapid. This work is an extension of the application of nano-enzyme catalysis in heavy metal ions analysis.

## Methods

### Instrument

A smart Raman spectrometer (DXR, Thermo, USA), a fluorescence spectrophotometer (F-7000, Hitachi High-Technologies Corporation, Japan), a double beam UV-visible spectrophotometer (TU-1901, Beijing Purkingje General Instrument Co., Ltd. China), a heating magnetic stirrer (79-1, Zhongda Instrumental Plant, Jiangsu, China), a electric hot water bath (HH-S2, Earth Automation Instrument Plant, Jintan, China), a nanometer particle size and zeta potential analyzer (Zetasizer Nano, Malvern, UK) and a microwave digestion instrument (WX-6000, PreeKem, Shanghai, China) were used^46^.

### Reagents

84 μmol/L HAuCl_4_, 0.1 mol/L H_2_O_2_, 50 mmol/L KOH, 50 mmol/L NaOH, 0.01 mol/L AgNO_3_, 6 mmol/L HCl, 1 µmol/L Pb(II), 30.85 μmol/L lead ion nucleic acid aptamer (Apt-Pb) sequence: 5′-3′GGT TGG TGT GGT TGG (Handsome Biotechnology Co., Ltd.), diluted 100 times to 0.3085 μmol/L Apt, 10 μmol/L Victoria blue B(VBB) solution, glucose (AR) and citric acid (AR) were prepared. The reagents and the water used above were analytical grade and ultrapure water respectively.

### Procedure

At room temperature, a 200 μL Apt (0.3085 μmol/L) reacted with a certain volume of Pb(II) for 10 min in a test tube. Then a 200 μL CD_Ca_/CD_Ag0-3_/CD_Au0-4_, 30 μL 0.1 mmol/L H_2_O_2_, 200 μL HAuCl_4_ (84 μmol/L) and 40 μL HCl (6 mmol/L) were mixed in order and diluted to1.5 mL. The mixture reacted 20 min in a 60 °C water bath before being stopped by cold water. After adding 40 μL VBB (10 μmol/L), the mixed solution was characterized by a Raman spectrometer. The SERS value *I* and the blank *I*_0_ (without analyte) were detected, so the difference value Δ*I* (I–I_0_) was achieved.

## Supplementary information

A dual-model SERS and RRS analytical platform for Pb(II) based on Ag-doped carbon dot catalytic amplification and aptamer regulation
