# Supplementary material for: A dual-model SERS and RRS analytical platform for Pb(II) based on Ag-doped carbon dot catalytic amplification and aptamer regulation
Source: Sci Rep. 2019 Jul 10;9:9991. doi: 10.1038/s41598-019-46426-y (PMC6620292; doi:10.1038/s41598-019-46426-y)
Supplement: Supplementary file 1 — A dual-model SERS and RRS analytical platform for Pb(II) based on Ag-doped carbon dot catalytic amplification and aptamer regulation [file 41598_2019_46426_MOESM1_ESM.doc]

**A dual-model SERS and RRS analytical platform for Pb(II) based on Ag-doped carbon dot catalytic amplification and aptamer regulation**

**Haidong Wang, Xiaowei Huang,** **Guiqing Wen^*^, Zhiliang Jiang***

Key Laboratory of Ecology of Rare and Endangered Species and Environmental Protection (Guangxi Normal University), Ministry of Education, Guangxi Key Laboratory of Environmental Pollution Control Theory and Technology, Guilin 541004, China

**Keywords:** Pb(II), Aptamer, carbon dots catalysis, SERS

**Preparation of carbon dots (CDs)**

**CD_Ca_:** In a 100 mL Teflon reactor, 10 mL of deionized water, 1 g of citric acid and 200 mg of Ca(OH)_2_ were added in sequence and the mixture was shake to dissolve. Then, 500 μL of ethylenediamine was slowly added, mixed evenly. Finally, it reacted in a Muffle furnace for 4 h with an optimum temperature of 200 ℃. We got a brown liquid solution, which was adjusted to neutrality with 50 mmol/L KOH and diluted to 20 mL. And then diluted 10 thousand times to a concentration of 1 μg/mL CD_Ca_. The CDs concentration was calculated in terms of the total amount of carbon added.

**CD_Ag_:** 1 g of glucose and a certain amount of (0, 500 μL, 2 mL, 3 mL) AgNO_3_ (0.01 mol/L) solution were dissolved in 20 mL of deionized water. Then, it was transferred into a Teflon reactor and reacted for 1 h at an optimum temperature of 160 ℃. We got a light yellow clear solution after the reaction was over. 1 mL of the obtained CD solution was adjusted to pH neutrality with 50 mmol/L KOH, and then diluted to 10 mL. The concentration of the CD, numbered CDAg_0-3_, was 1.8 mg/mL in terms of the total amount of carbon added.

**CD_Au_:** 1 g of glucose and a certain amount of (0, 1.0 mL, 1.5 mL, 2.0 mL, 3.0 mL) HAuCl_4_ (84 μmol/L) solution were dissolved in 20 mL of deionized water. Then, it was transferred into a Teflon reactor and reacted for 5 h at an optimum temperature of 180 ℃. We get a brownish yellow clear solution after the reaction is over. The solution was dialyzed for 12 h with a dialysis bag with a molecular weight cut off of 3500 Da. The concentration of the CD, numbered CD_Au0-4_, was 18 mg/mL in terms of the total amount of carbon added.


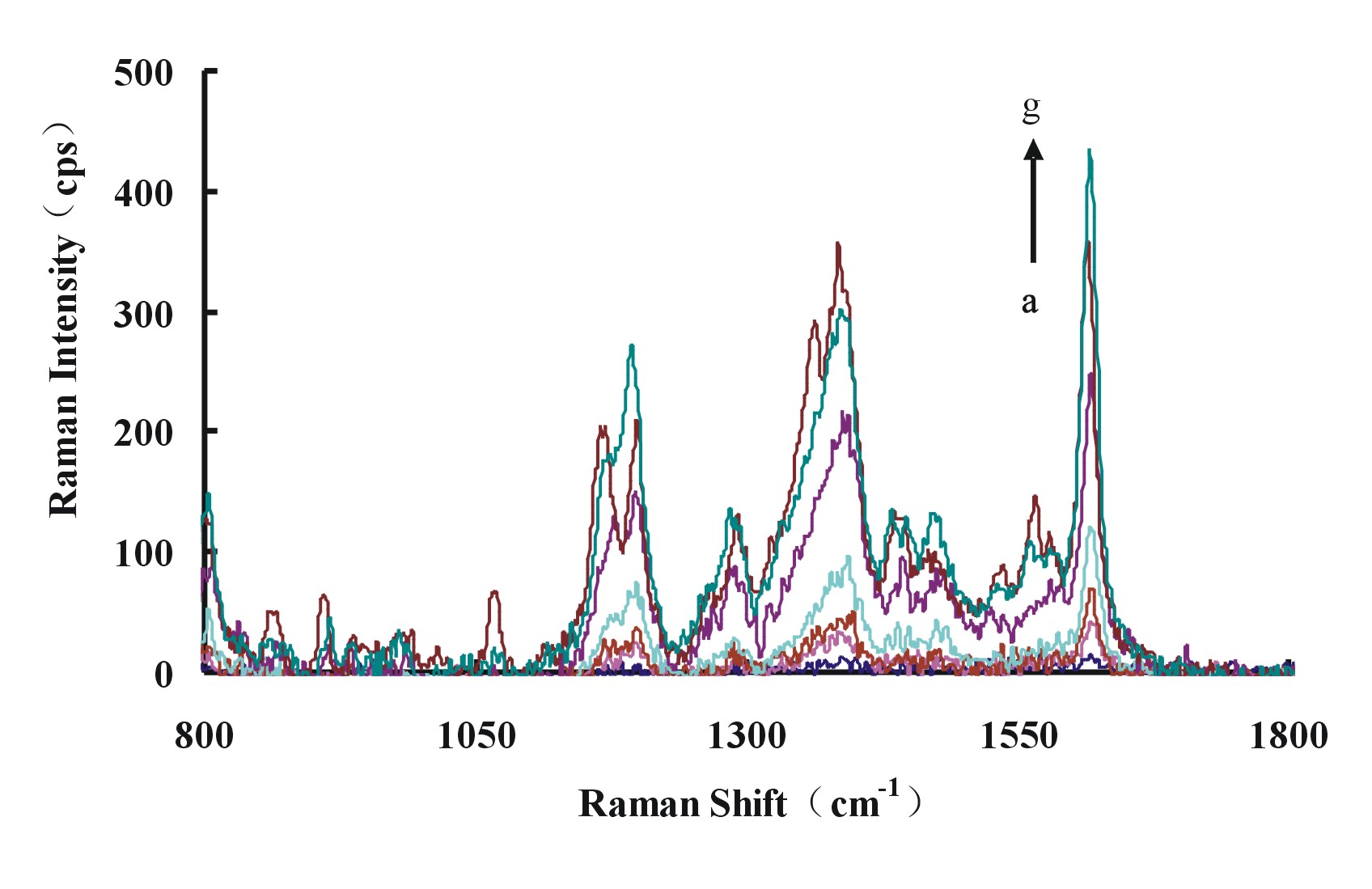


**Fig. S1 SERS spectra of CD_Ca_-HAuCl_4_-H_2_O_2_- HCl-VBB system**

a: 11.2 μmol/L HAuCl_4_+2 mmol/L H_2_O_2_+0.16 mmol/L HCl+0.27 μmol/L VBB; b: a+3.3 ng/mL CD_Ca_; c: a+6.67 ng/mL CD_Ca_; d: a+13.3 ng/mL CD_Ca_; e: a+20 ng/mL CD_Ca_; f: a+26.7 ng/mL CD_Ca_; g: a+30 ng/mL CD_Ca_


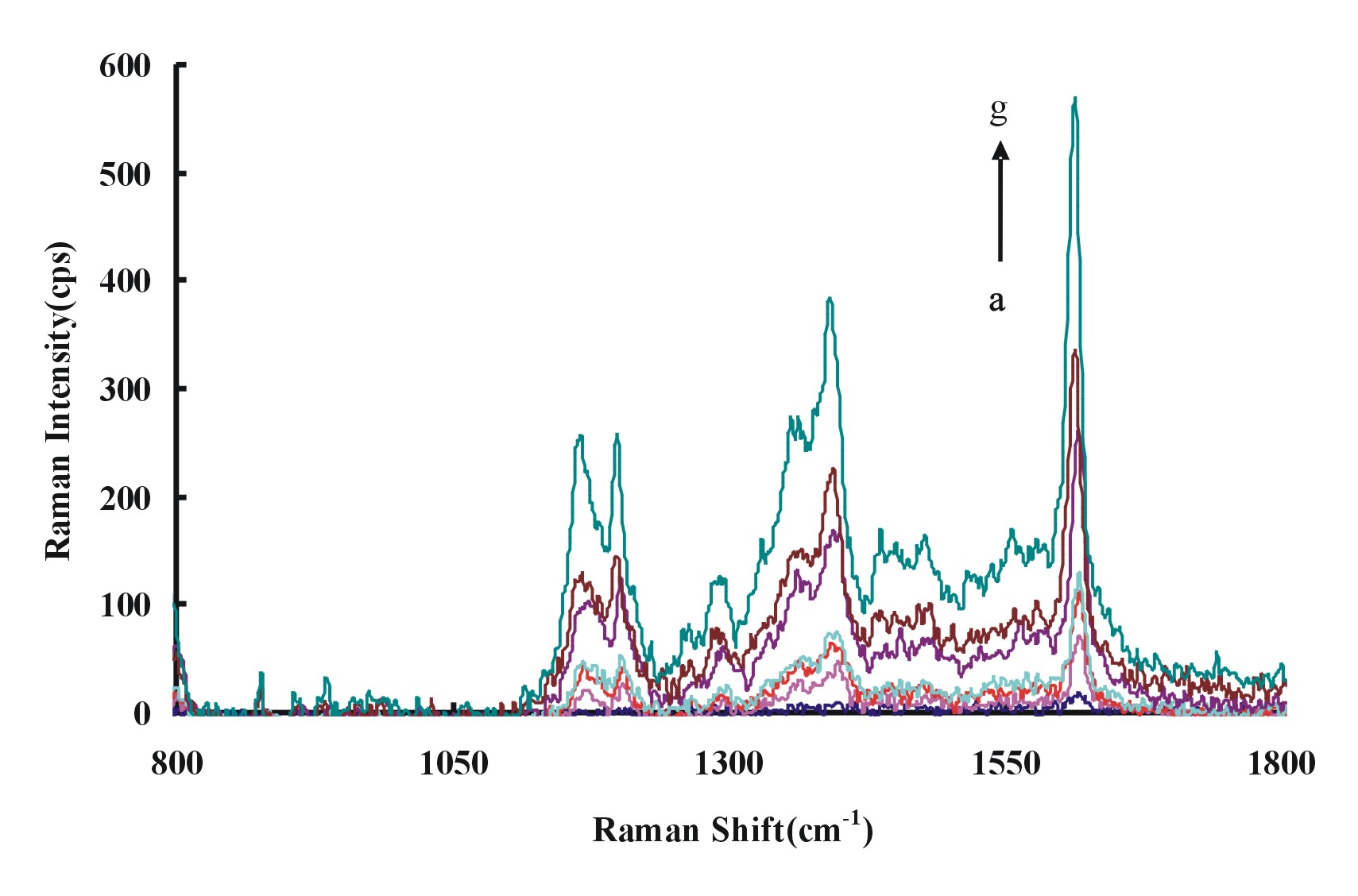


**Fig. S2 SERS spectra of CD_Ag0_-HAuCl_4_-H_2_O_2_- HCl-VBB system**

a: 0.16 mmol/L HCl+11.2 μmol/L HAuCl_4_+2 mmol/L H_2_O_2_+0.27 μmol/L VBB; b: a+0.06 mg/mL CD_Ag0_; c: a+0.12 mg/mL CD_Ag0_; d: a+0.18 mg/mL CD_Ag0_; e: a+0.24mg/mL CD_Ag0_ ; f: a+0.30 mg/mL CD_Ag0_; g: a+0.36 mg/mL CD_Ag0_.


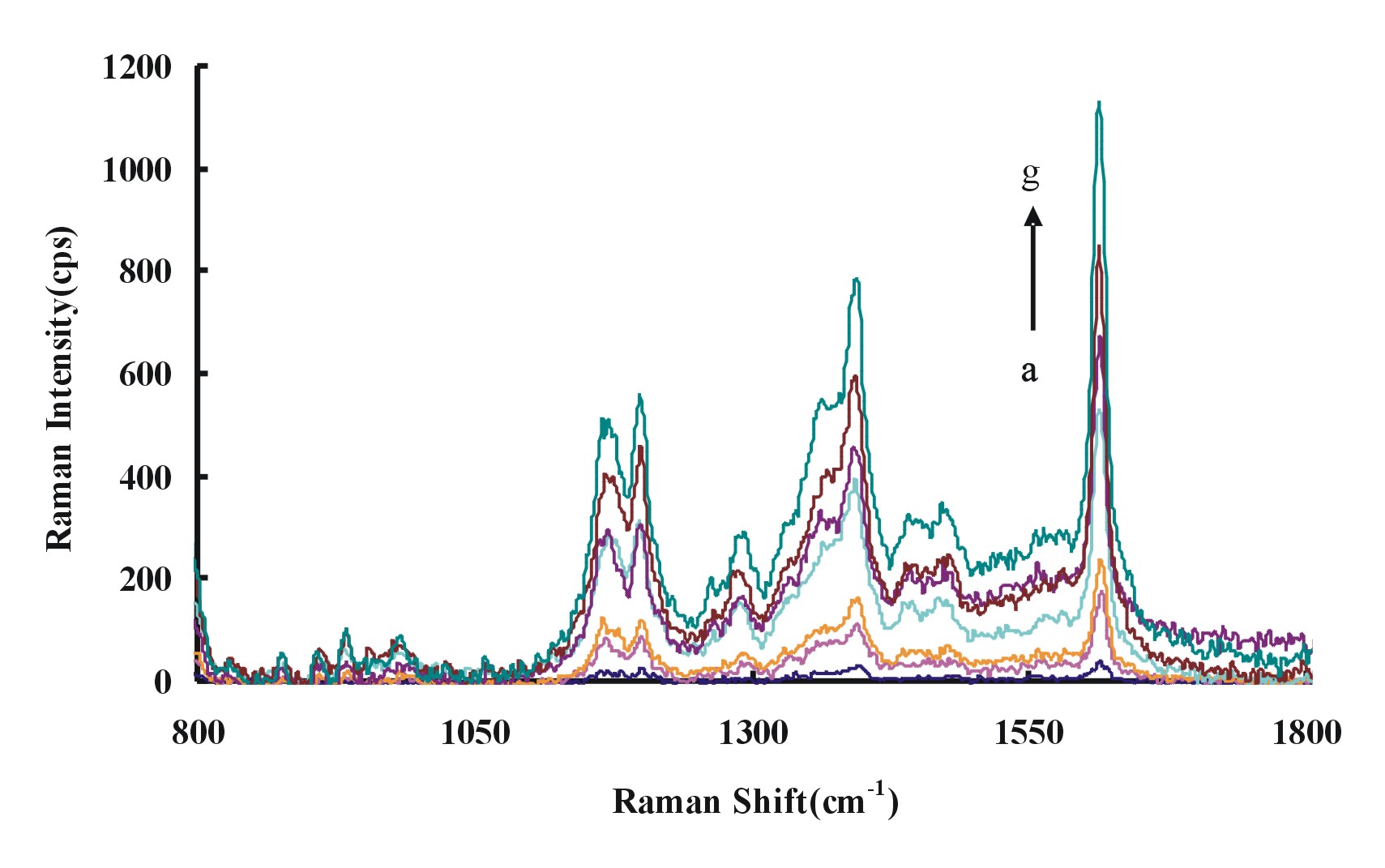


**Fig. S3 SERS spectra of CD_Ag1_-HAuCl_4_-H_2_O_2_- HCl-VBB system**

a: 0.16 mmol/L HCl+11.2 μmol/L HAuCl_4_+2 mmol/L H_2_O_2_+0.27 μmol/L VBB; b: a+0.04 mg/mL CD_Ag1_; c: a+0.06 mg/mL CD_Ag1_; d: a+0.12 mg/mL CD_Ag1_; e: a+0.18 mg/mL CD_Ag1_ ; f: a+0.24 mg/mL CD_Ag1_; g: a+0.36 mg/mL CD_Ag1_.


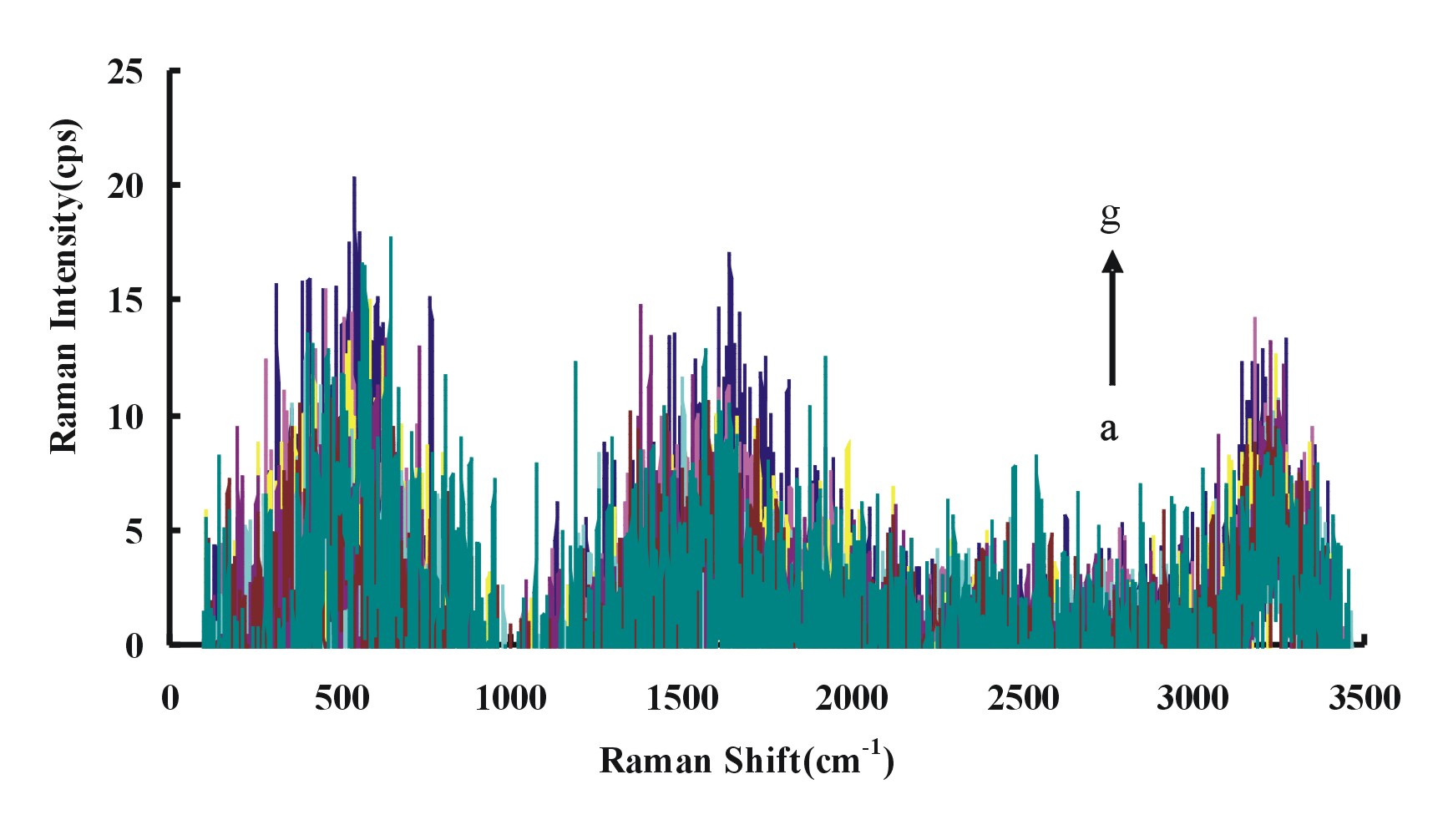


**Fig. S4 SERS spectra of CD_Ag1-3_-HAuCl_4_-H_2_O_2_- HCl-VBB（not heated）system**

a: 0.16 mmol/L HCl+11.2 μmol/L HAuCl_4_+2 mmol/L H_2_O_2_+0.27 μmol/L VBB; b: a+0.04 mg/mL CD_Ag1_; c: a+0.06 mg/mL CD_Ag1_; d: a+0.12 mg/mL CD_Ag1_; e: a+0.18 mg/mL CD_Ag1_; f: a+0.24 mg/mL CD_Ag1_; g: a+0.36 mg/mL CD_Ag1_.


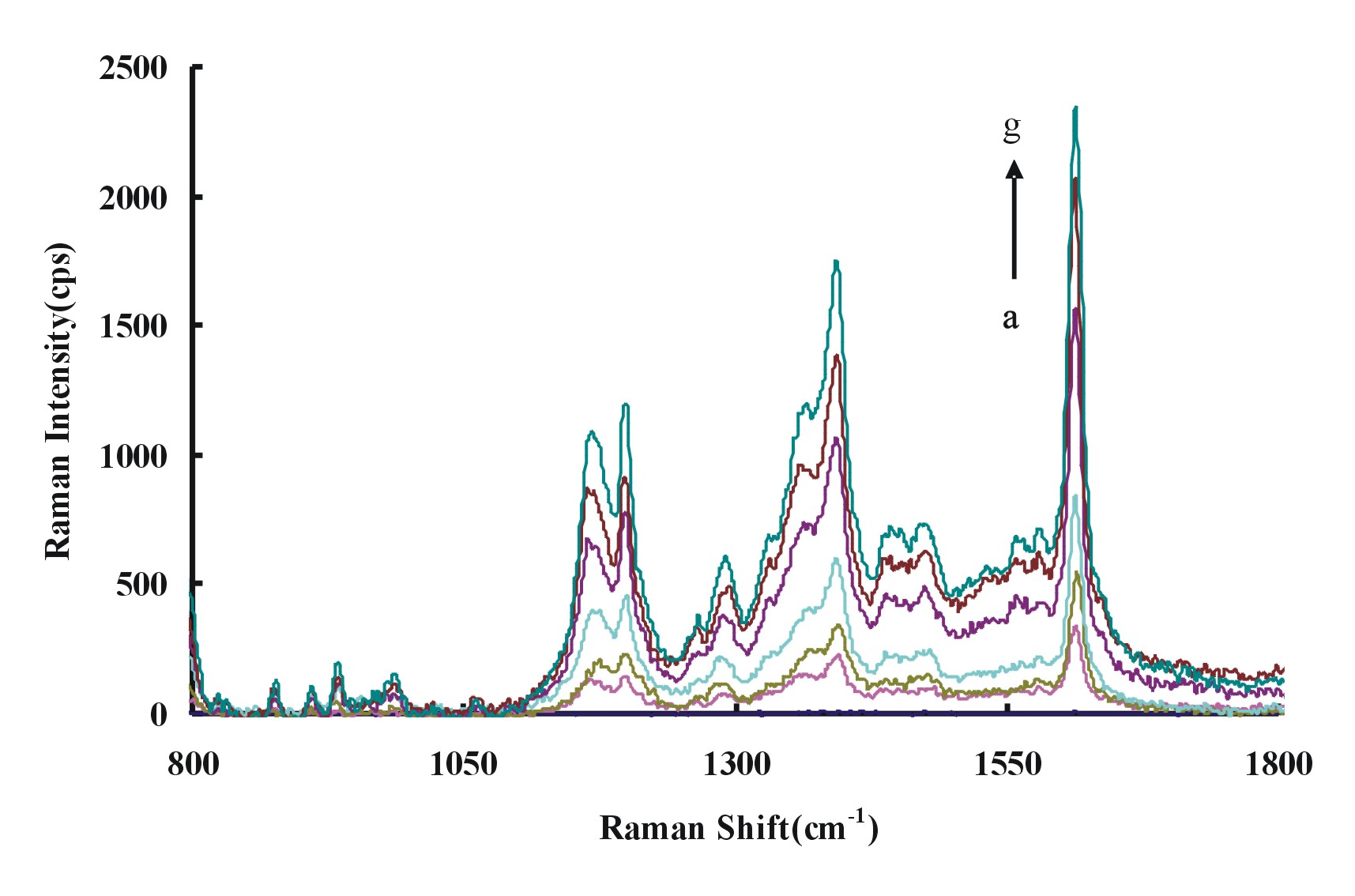


**Fig. S5 SERS spectra of CD_Ag2_-HAuCl_4_-H_2_O_2_- HCl-VBB system**

a: 0.16 mmol/L HCl+11.2 μmol/L HAuCl_4_+2 mmol/L H_2_O_2_+0.27 μmol/L VBB; b: a+0.02 mg/mL CD_Ag2_; c: a+0.06 mg/mL CD_Ag2_; d: a+0.12 mg/mL CD_Ag2_; e: a+0.24 mg/mL CD_Ag2_; f: a+0.36 mg/mL CD_Ag2_; g: a+0.48 mg/mL CD_Ag2_.


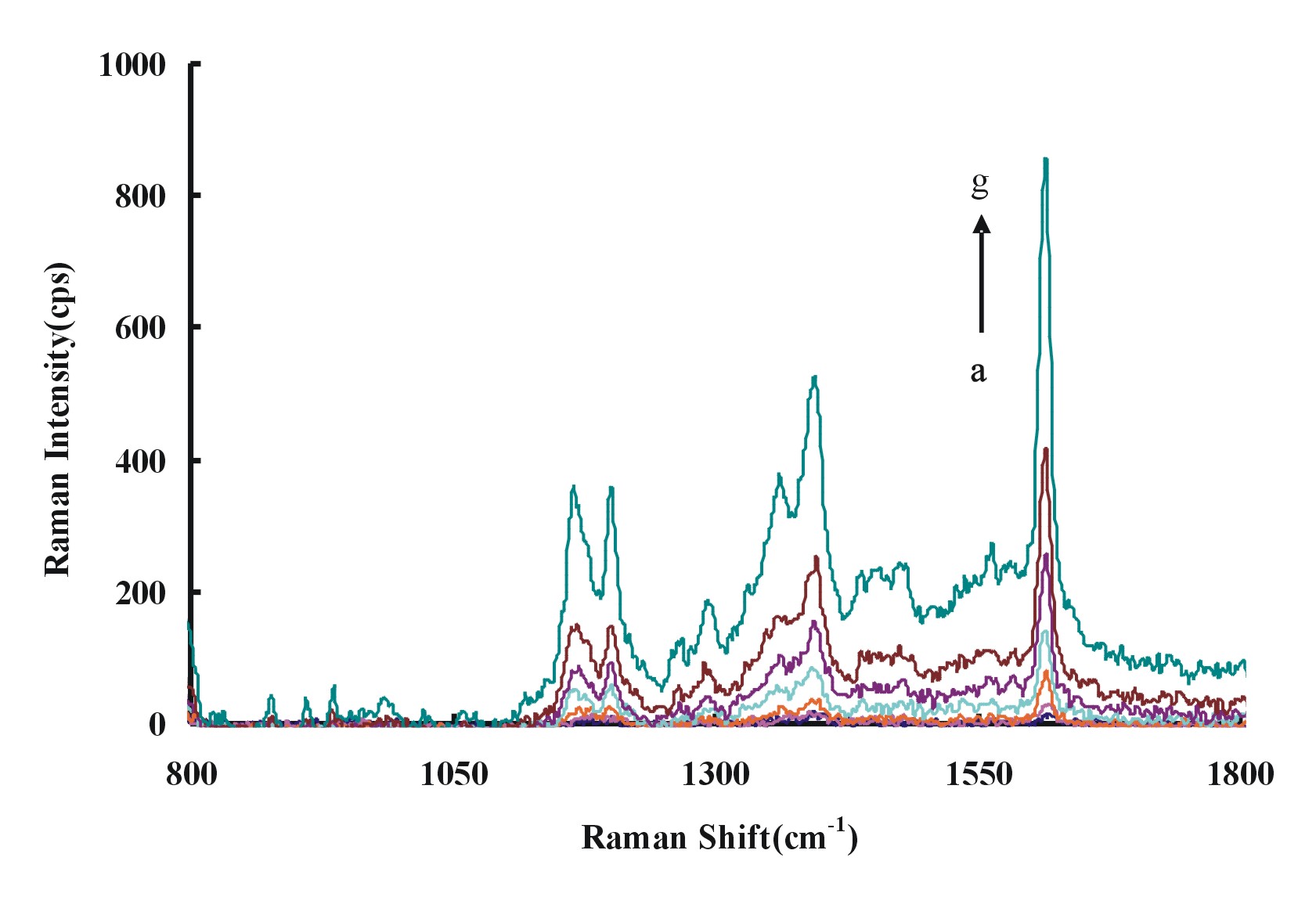


**Fig. S6 SERS spectra of CD_Ag3_-HAuCl_4_-H_2_O_2_- HCl-VBB system**

a: 0.16 mmol/L HCl+11.2 μmol/L HAuCl_4_+2 mmol/L H_2_O_2_+0.27 μmol/L VBB; b: a+0.04 mg/mL CD_Ag3_; c: a+0.06 mg/mL CD_Ag3_; d: a+0.12 mg/mL CD_Ag3_; e: a+0.18 mg/mL CD_Ag3_ ; f: a+0.24 mg/mL CD_Ag3_; g: a+0.36 mg/mL CD_Ag3_.

**
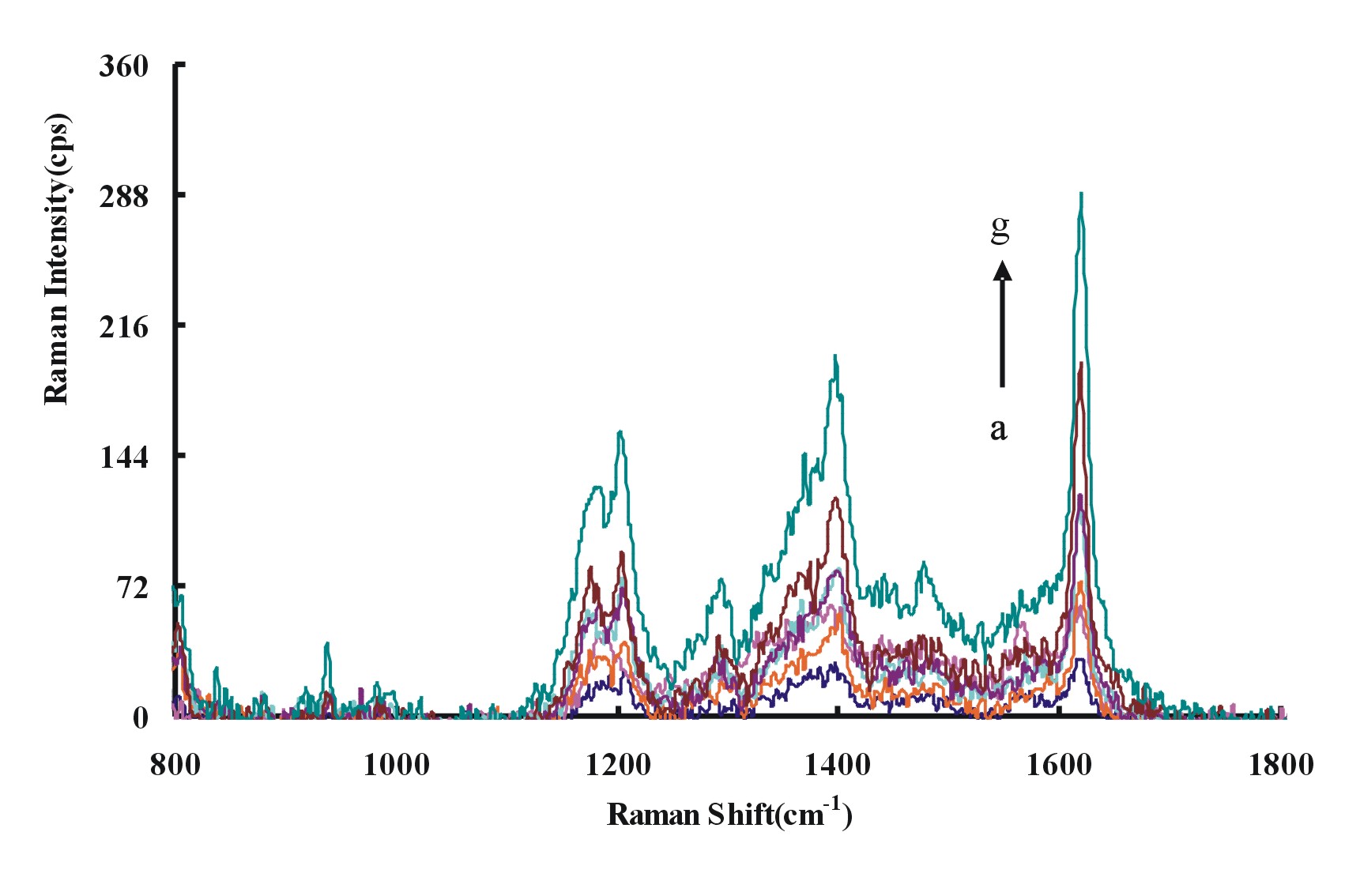
**

**Fig. S7 SERS spectra of CD_Au0_-HAuCl_4_-H_2_O_2_- HCl-VBB system**

a: 0.16 mmoL/L HCl+11.2 μmol/L HAuCl_4_+2 mmol/L H_2_O_2_+0.27μmol/L VBB; b: a+0.6 mg/mL CD_Au0_; c: a+1.2 mg/mL CD_Au0_; d: a+2.4 mg/mL CD_Au0_; e: a+3.6 mg/mL CD_Au0_; f: a+4.8 mg/mL CD_Au0_; g: a+6 mg/mL CD_Au0_.


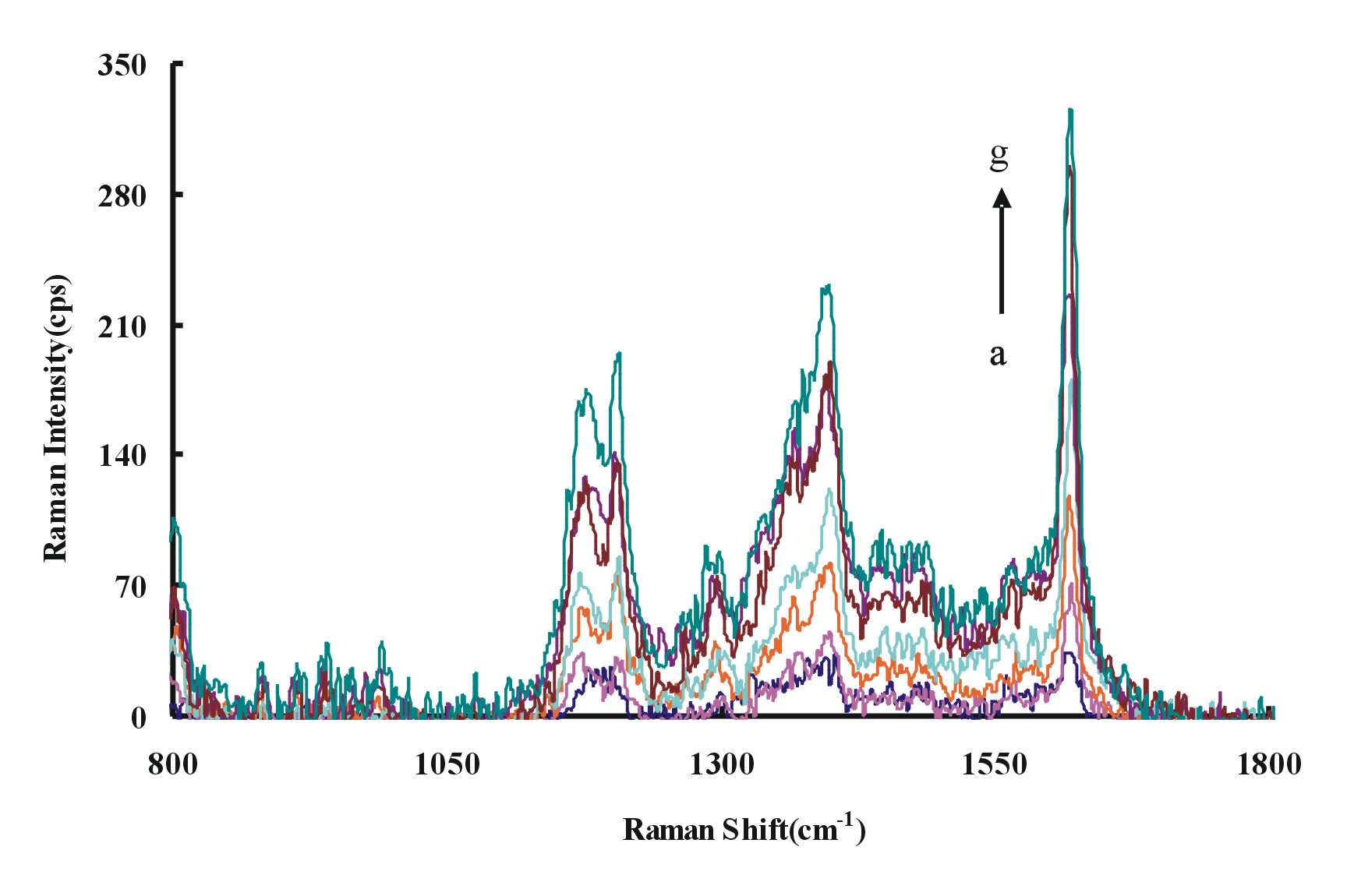


**Fig. S8 SERS spectra of CD_Au1_-HAuCl_4_-H_2_O_2_- HCl-VBB system**

a: 0.16 mmol/L HCl+11.2 μmol/L HAuCl_4_+2 mmol/L H_2_O_2_+0.27 μmol/L VBB; b: a+0.6 mg/mL CD_Au1_; c: a+1.2 mg/mL CD_Au1_; d: a+2.4 mg/mL CD_Au1_; e: a+3.6 mg/mL CD_Au1_; f: a+4.8 mg/mL CD_Au1_; g: a+6 mg/mL CD_Au1_.


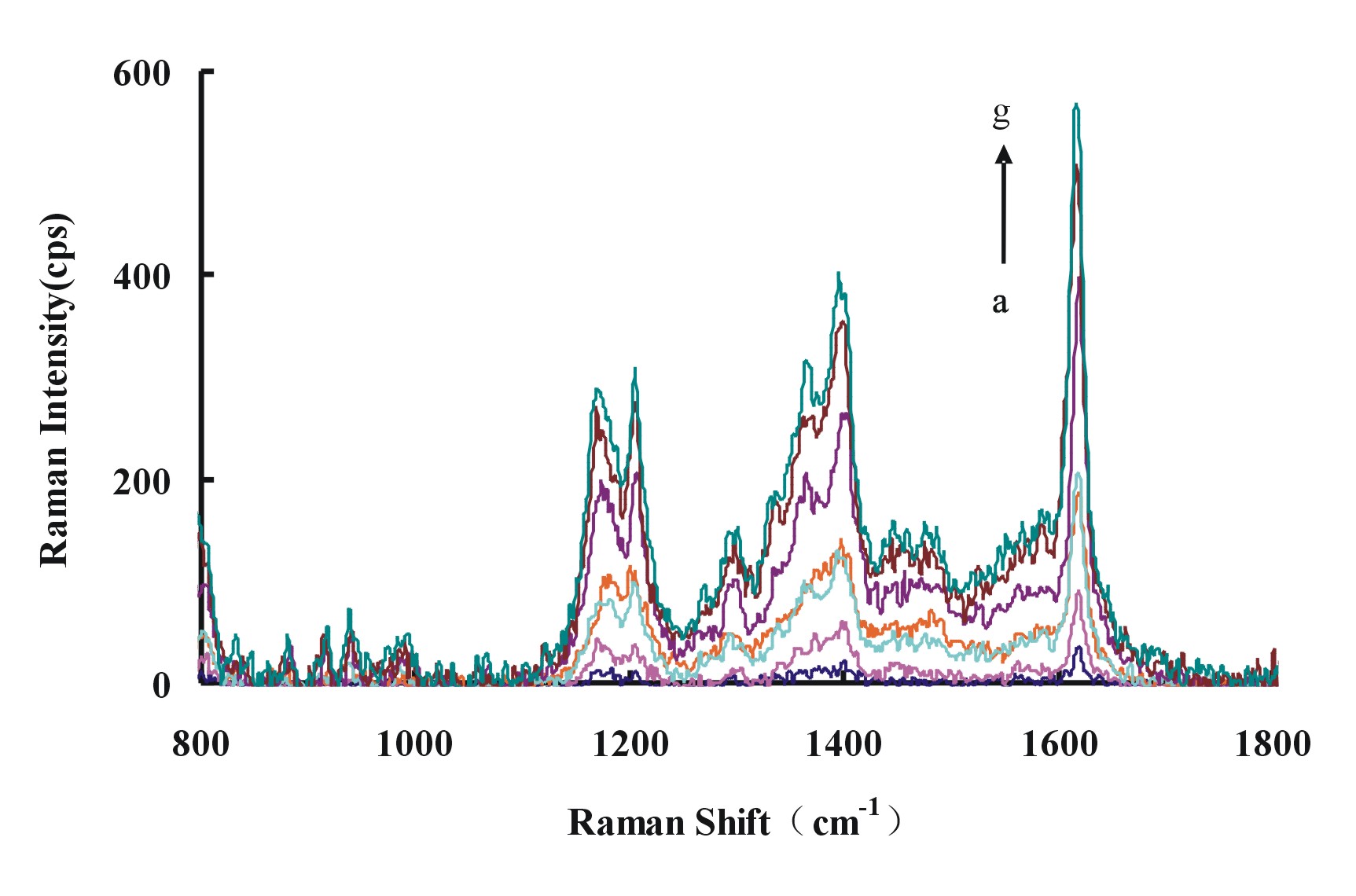


**Fig. S9 SERS spectra of CD_Au2_-HAuCl_4_-H_2_O_2_- HCl-VBB system**

a: 0.16 mmol/L HCl+11.2 μmol/L HAuCl_4_+2 mmol/L H_2_O_2_+0.27 μmol/L VBB; b: a+0.6 mg/mL CD_Au2_; c: a+1.2 mg/mL CD_Au2_; d: a+2.4 mg/mL CD_Au2_; e: a+3.6 mg/mL CD_Au2_; f: a+4.8 mg/mL CD_Au2_; g: a+6 mg/mL CD_Au2_.


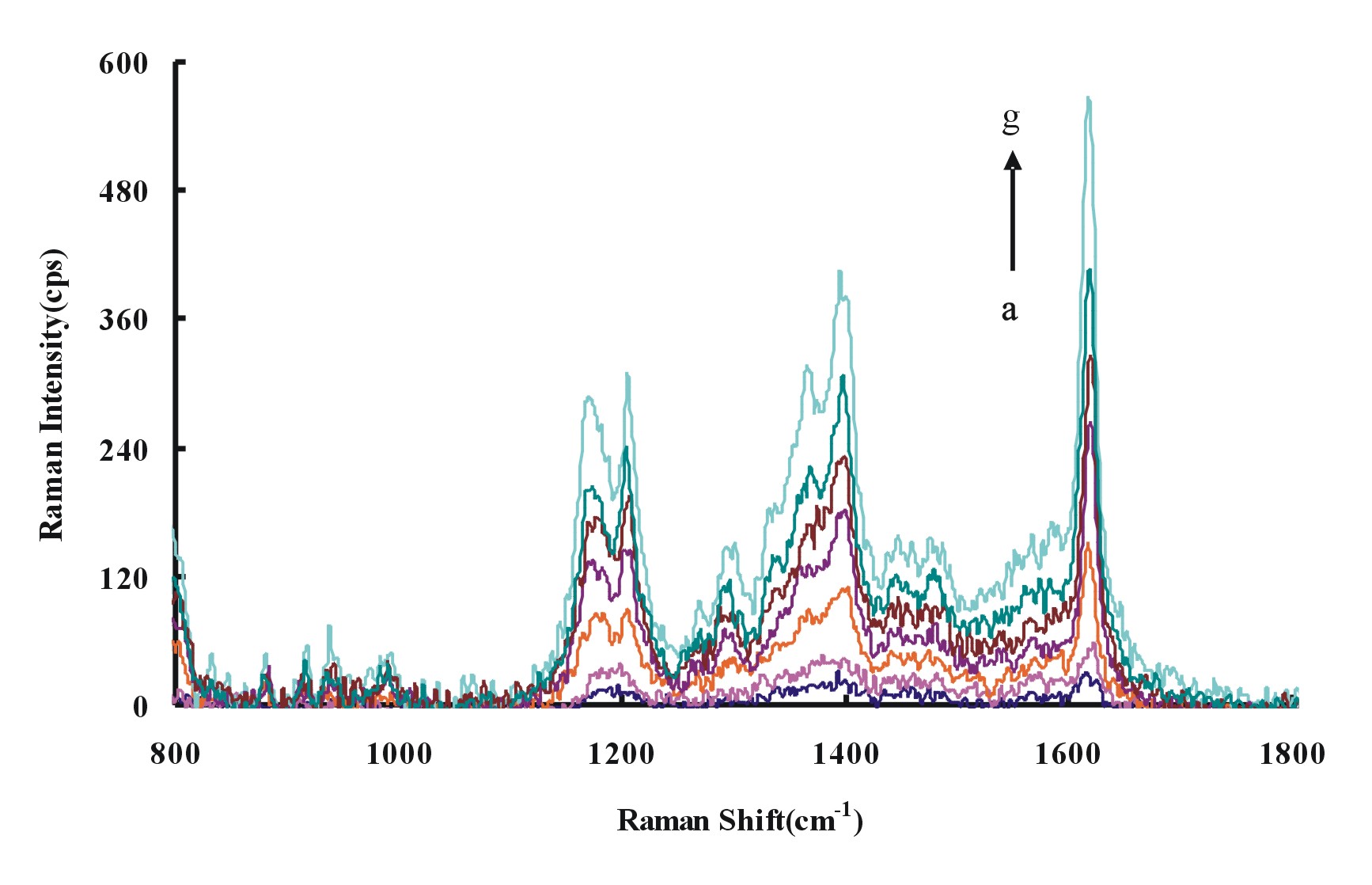


**Fig. S10 SERS spectra of CD_Au3_-HAuCl_4_-H_2_O_2_- HCl-VBB system**

a: 0.16 mmol/L HCl+11.2 μmol/L HAuCl_4_+2 mmol/L H_2_O_2_+0.27 μmol/L VBB; b: a+0.6 mg/mL CD_Au3_; c: a+1.2 mg/mL CD_Au3_; d: a+2.4 mg/mL CD_Au3_; e: a+3.6 mg/mL CD_Au3_; f: a+4.8 mg/mL CD_Au3_; g: a+6 mg/mL CD_Au3_.

**
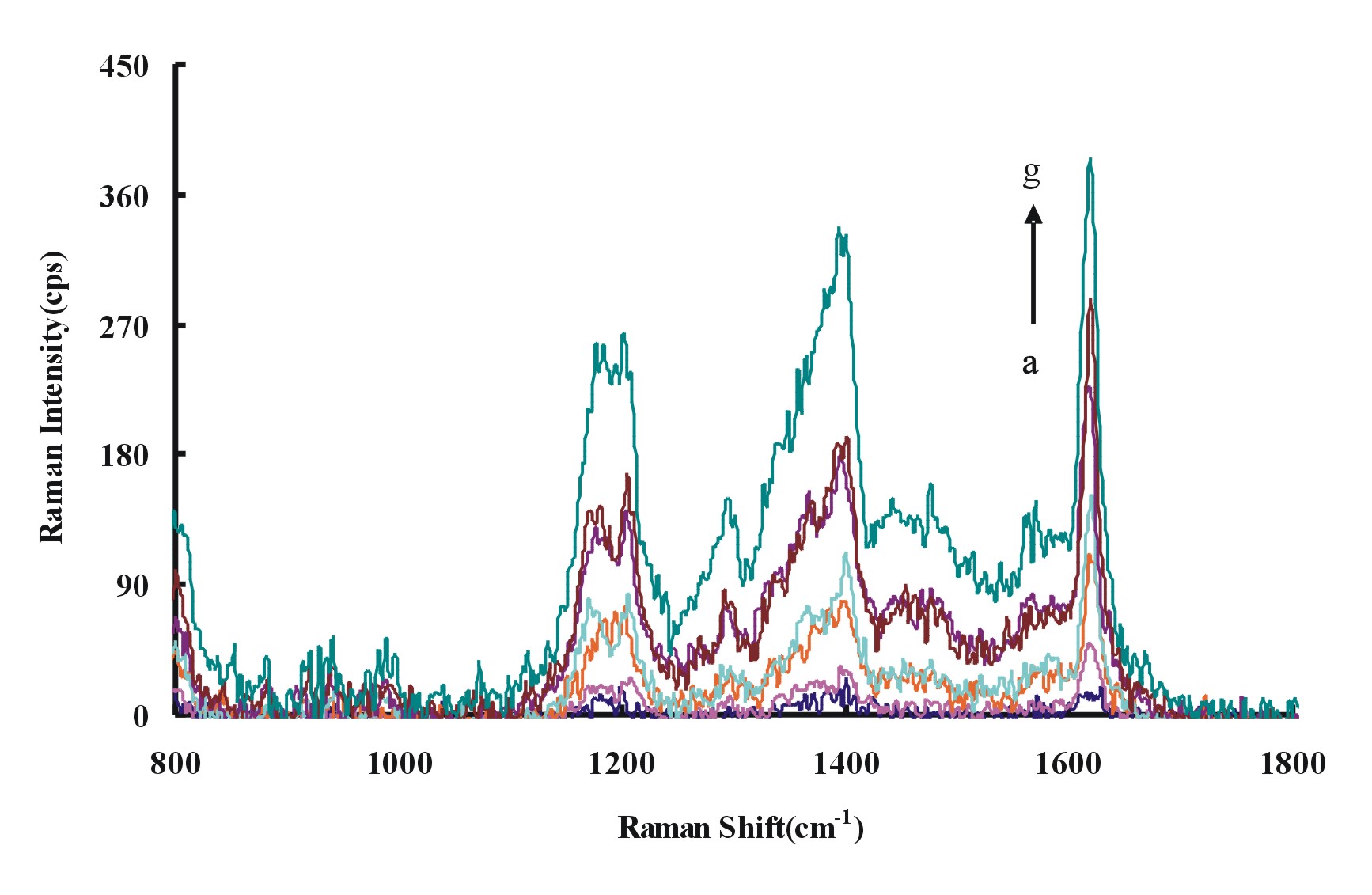
**

**Fig. S11 SERS spectra of CD_Au4_-HAuCl_4_-H_2_O_2_- HCl-VBB system**

a: 0.16 mmol/L HCl+11.2 μmol/L HAuCl_4_+2 mmol/L H_2_O_2_+0.27 μmol/L VBB; b: a+0.6 mg/mL CD_Au4_; c: a+1.2 mg/mL CD_Au4_; d: a+2.4 mg/mL CD_Au4_; e: a+3.6 mg/mL CD_Au4_; f: a+4.8 mg/mL CD_Au4_; g: a+6 mg/mL CD_Au4_.


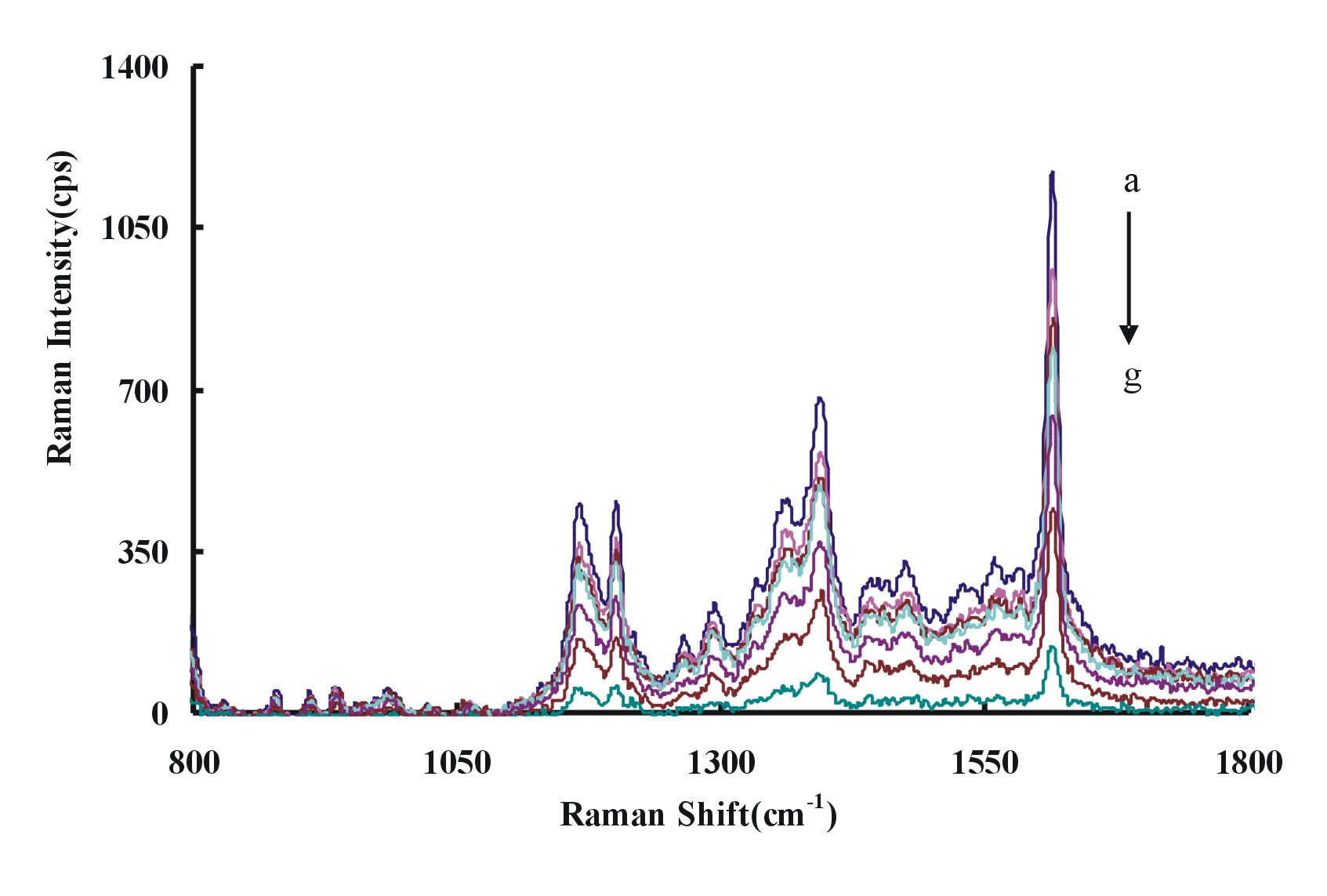


**Fig. S12 SERS spectra of Apt-CD_Ag2_-HAuCl_4_-H_2_O_2_-HCl-VBB system**

a: 0.36 mg/mL CD_Ag2_+ 0.16 mmol/L HCl+11.2 μmol/L HAuCl_4_+2 mmol/L H_2_O_2_+0.27 μmol/L VBB; b:a+1 nmol/L Apt; c: a+2 nmol/L Apt; d: a+4 nmol/L Apt; e: a+6 nmol/L Apt ; f: a+8 nmol/L Apt; g: a+10 nmol/L Apt.


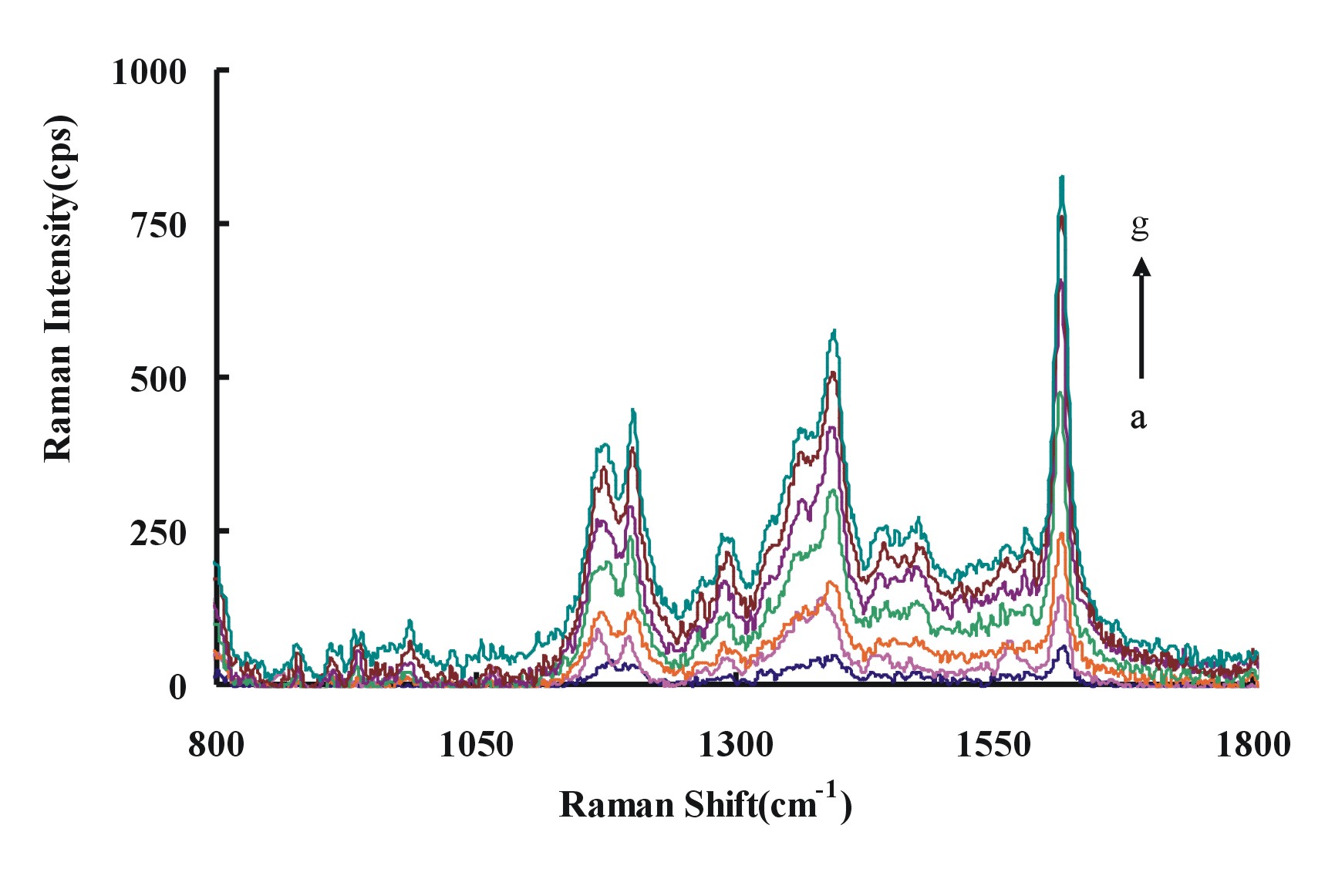


**Fig. S13 SERS spectra of Apt-** **Pb(II)-CD_Ca_-HAuCl_4_-H_2_O_2_-VBB system**

a: 40 nmol/L Apt+ 0.36 mg/mL CD_Ag0_+0.16 mmol/L HCl+11.2 μmol/L HAuCl_4_+2 mmol/L H_2_O_2_+0.27 μmol/L VBB; b:a+0.02 μmol/L Pb(II); c:a+0.17 μmol/L Pb(II); d: a+0.3 μmol/L Pb(II); e: a+0.43 μmol/L Pb(II); f:a+0.57 μmol/L Pb(II); g:a+0.67 μmol/L Pb(II)


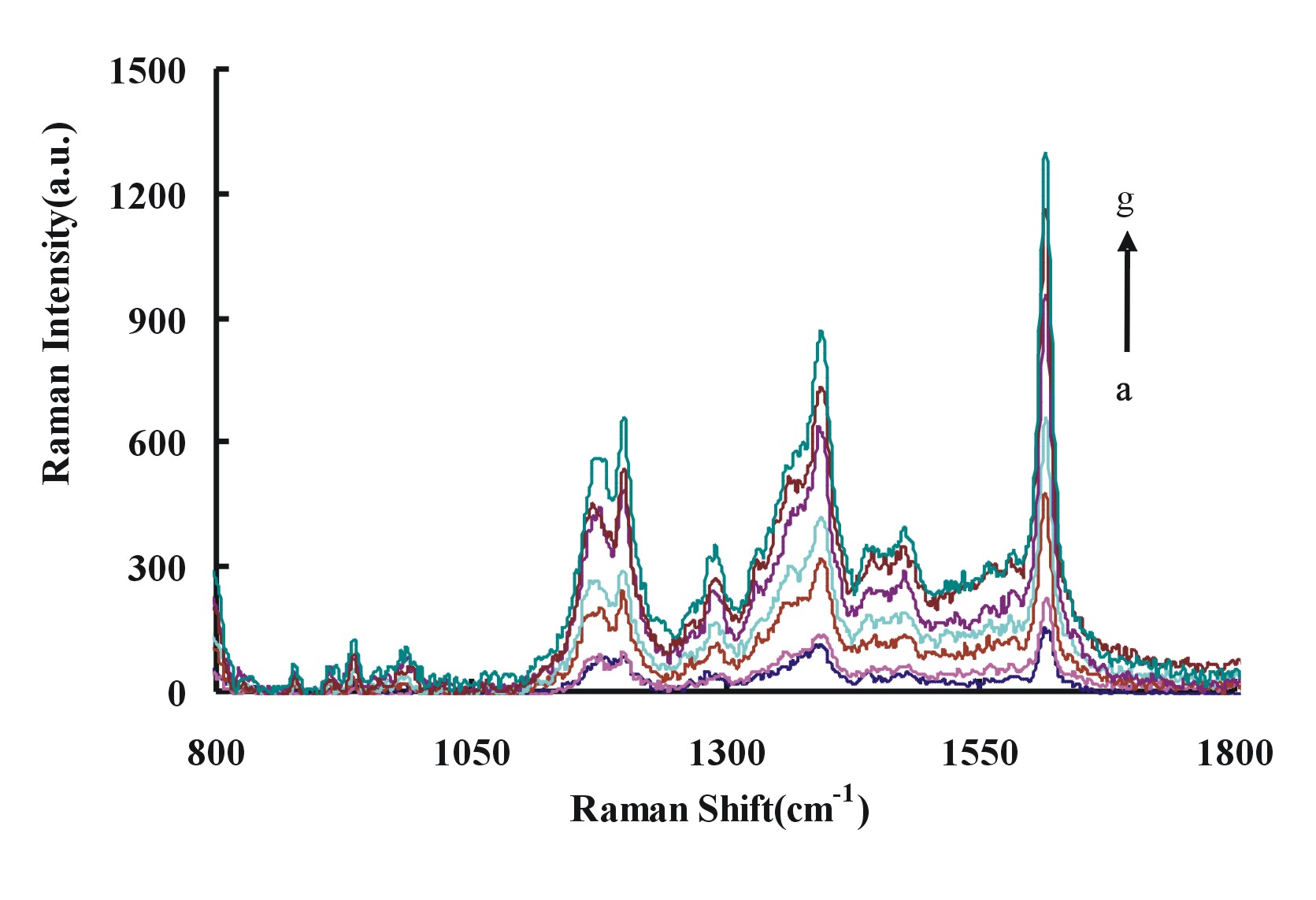


**Fig. S14 SERS spectra of Apt- Pb(II)-CD_Ag0_-HAuCl_4_-H_2_O_2_-HCl-VBB system**

a: 40 nmol/L Apt+ 0.36 mg/mL CD_Ag0_+0.16 mmol/L HCl+11.2μmol/L HAuCl_4_+2 mmol/L H_2_O_2_+0.27 μmol/L VBB; b:a+0.013 μmol/L Pb(II); c:a+0.1 μmol/L Pb(II); d: a+0.2 μmol/L Pb(II); e: a+0.27 μmol/L Pb(II); f:a+0.33 μmol/L Pb(II); g:a+0.46 μmol/L Pb(II)


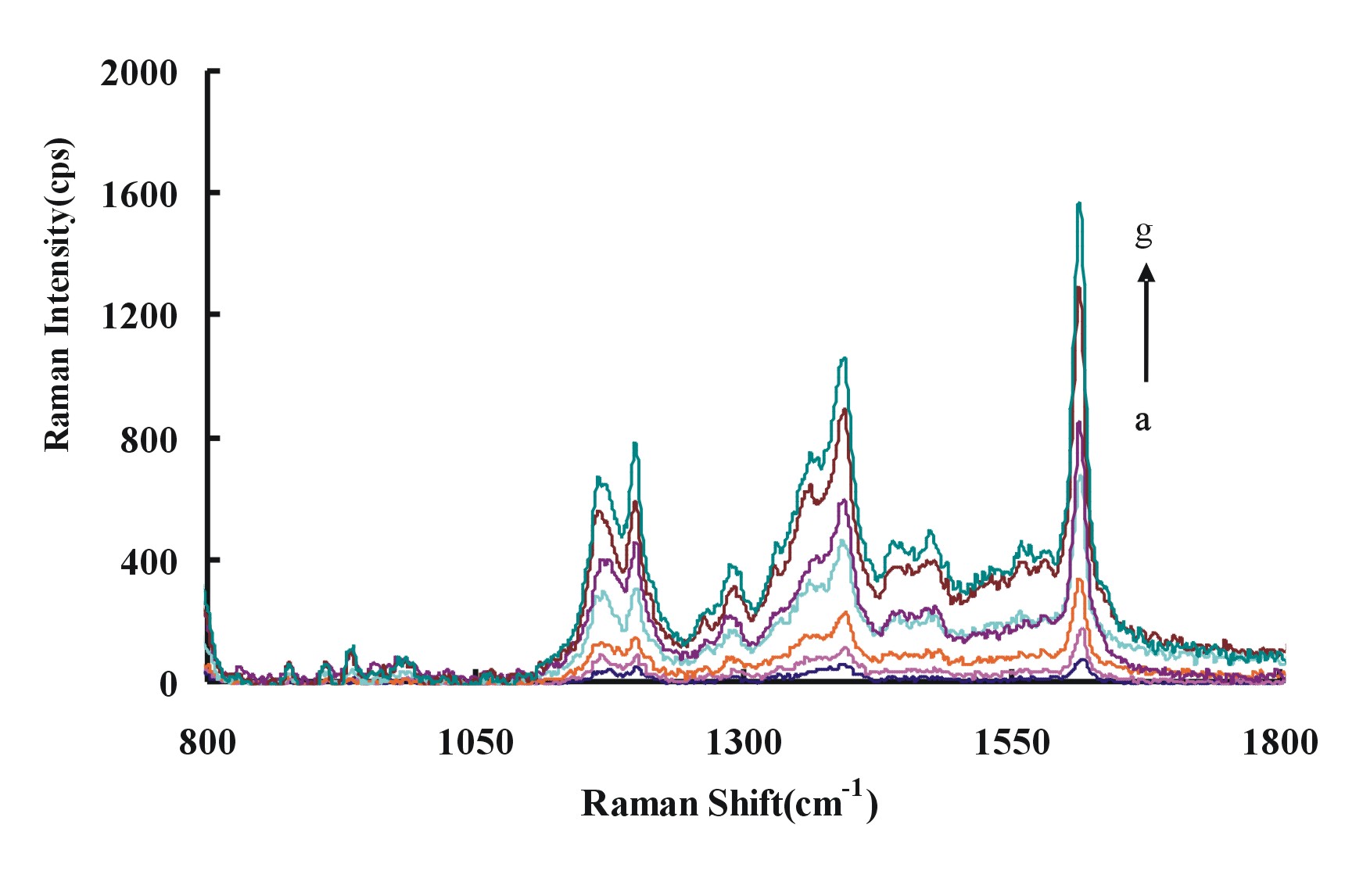


**Fig. S15 SERS spectra of Apt- Pb(II)-CD_Ag1_-HAuCl_4_-H_2_O_2_-HCl-VBB system**

a: 40 nmol/L Apt+ 0.36 mg/mL CD_Ag2_+0.16 mmol/L HCl+11.2μmol/L HAuCl_4_+2 mmol/L H_2_O_2_+0.27 μmol/L VBB; b:a+0.013 μmol/L Pb(II); c:a+0.026 μmol/L Pb(II); d: a+0.067 μmol/L Pb(II); e: a+0.13 μmol/L Pb(II); f:a+0.27 μmol/L Pb(II); g:a+0.46 μmol/L Pb(II)


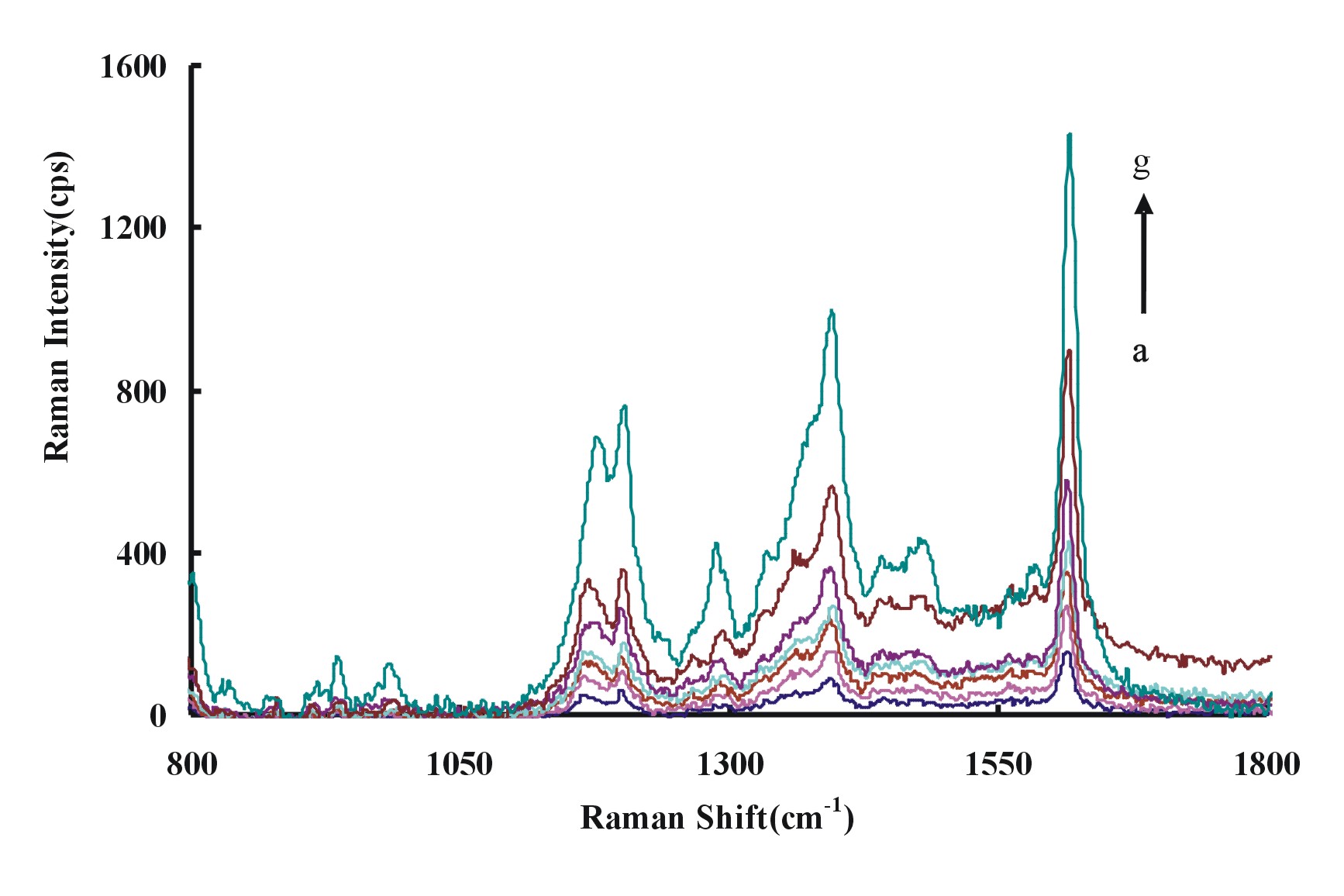


**Fig. S16 SERS spectra of Apt-Pb(II)-CD_Ag3_-HAuCl_4_-H_2_O_2_- HCl-VBB system**

a: 80 nmol/L Apt+ 0.36 mg/mL CD_Ag3_+0.16 mmol/L HCl+11.2 μmol/L HAuCl_4_+2 mmol/L H_2_O_2_+0.27 μmol/L VBB; b:a+0.026 μmol/L Pb(II); c:a+0.067 μmol/L Pb(II); d: a+0.13 μmol/L Pb(II); e: a+0.2 μmol/L Pb(II); f:a+0.33 μmol/L Pb(II); g:a+0.53 μmol/L Pb(II)


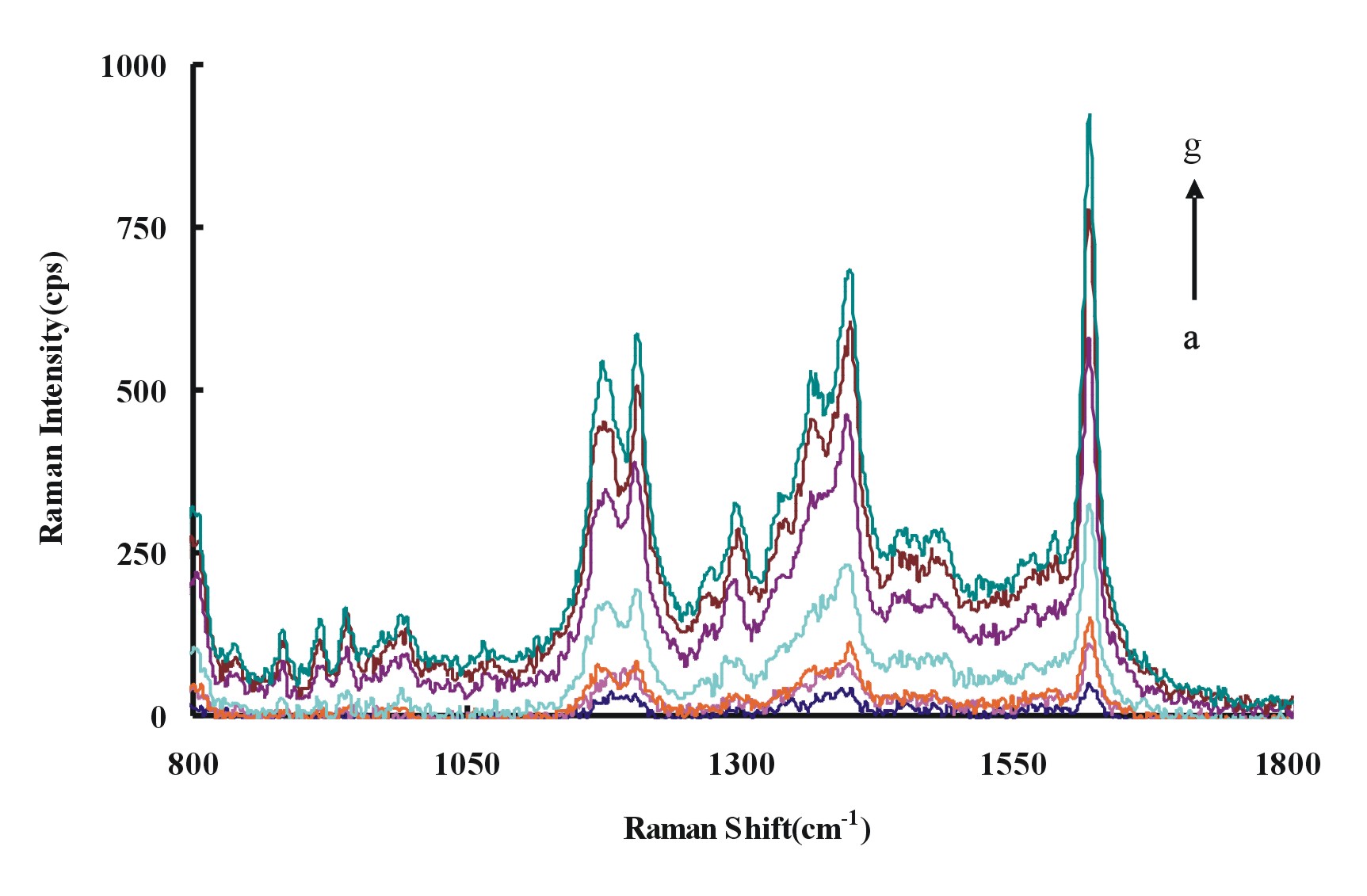


**Fig. S17 SERS spectra of Apt-Pb(II)-CD_Au3_-HAuCl_4_-H_2_O_2_-HCl-VBB system**

a: 80 nmol/L Apt+ 0.36 mg/mL CD_Au3_+0.16 mmol/L HCl+11.2 μmol/L HAuCl_4_+2 mmol/L H_2_O_2_+0.27 μmol/L VBB; b:a+0.026 μmol/L Pb(II); c:a+0.067 μmol/L Pb(II); d: a+0.13 μmol/L Pb(II); e: a+0.2 μmol/L Pb(II); f:a+0.33 μmol/L Pb(II); g:a+0.53 μmol/L Pb(II)


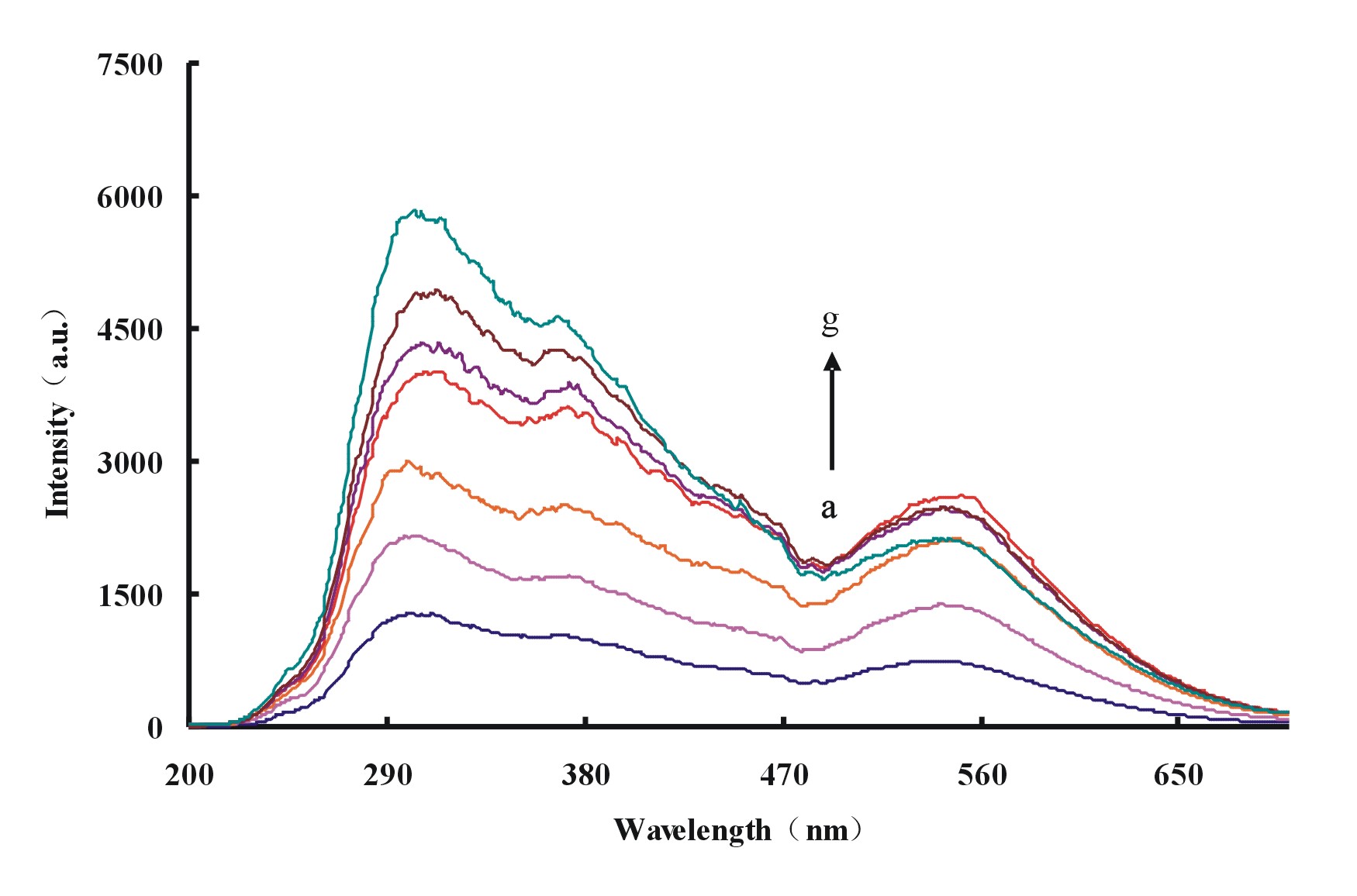


**Fig. S18 RRS spectra of CD_Ag2_-HAuCl_4_-H_2_O_2_-HCl system**

a: 0.16 mmol/L HCl +11.2 μmol/L HAuCl_4_+2 mmol/L H_2_O_2_; b: a+0.04 mg/mL CD_Ag2_; c: a+0.14 mg/mL CD_Ag2_; d: a+0.25 mg/mL CD_Ag2_; e: a+0.39 mg/mL CD_Ag2_; f: a+0.42 mg/mL CD_Ag2_; g: a+0.59 mg/mL CD_Ag2_.


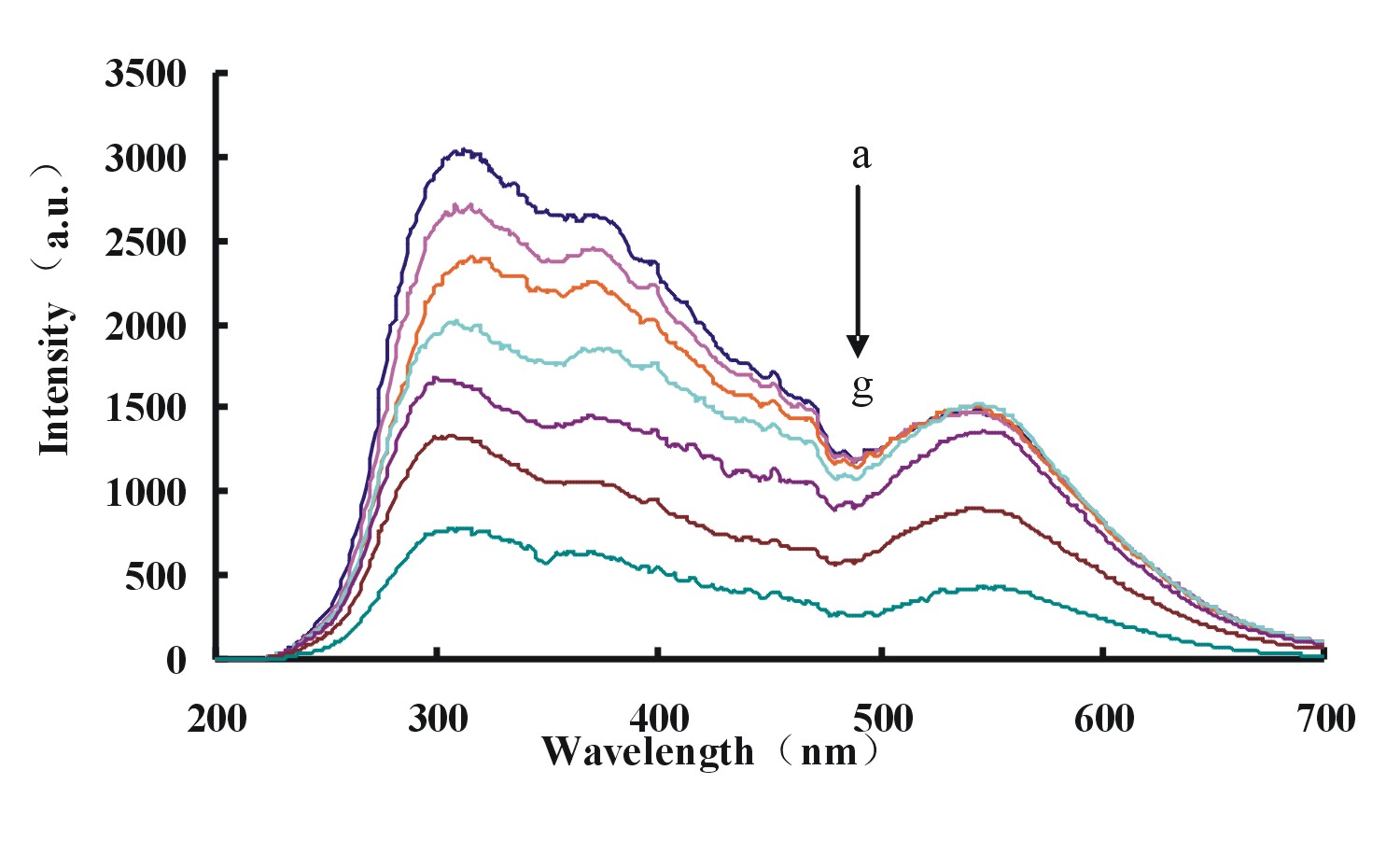


**Fig. S19 RRS spectra of Apt-CD_Ag2_-HAuCl_4_-H_2_O_2_-HCl system**

a: 0.36 mg/mL CD_Ag2_+0.16 mmol/L HCl + 11.2 μmol/L HAuCl_4_+2 mmol/L H_2_O_2_; b:a+3 nmol/L Apt; c: a+20 nmol/L Apt; d: a+40 nmol/L Apt; e: a+60 nmol/L Apt ; f: a+80 nmol/L Apt; g: a+200 nmol/L Apt.


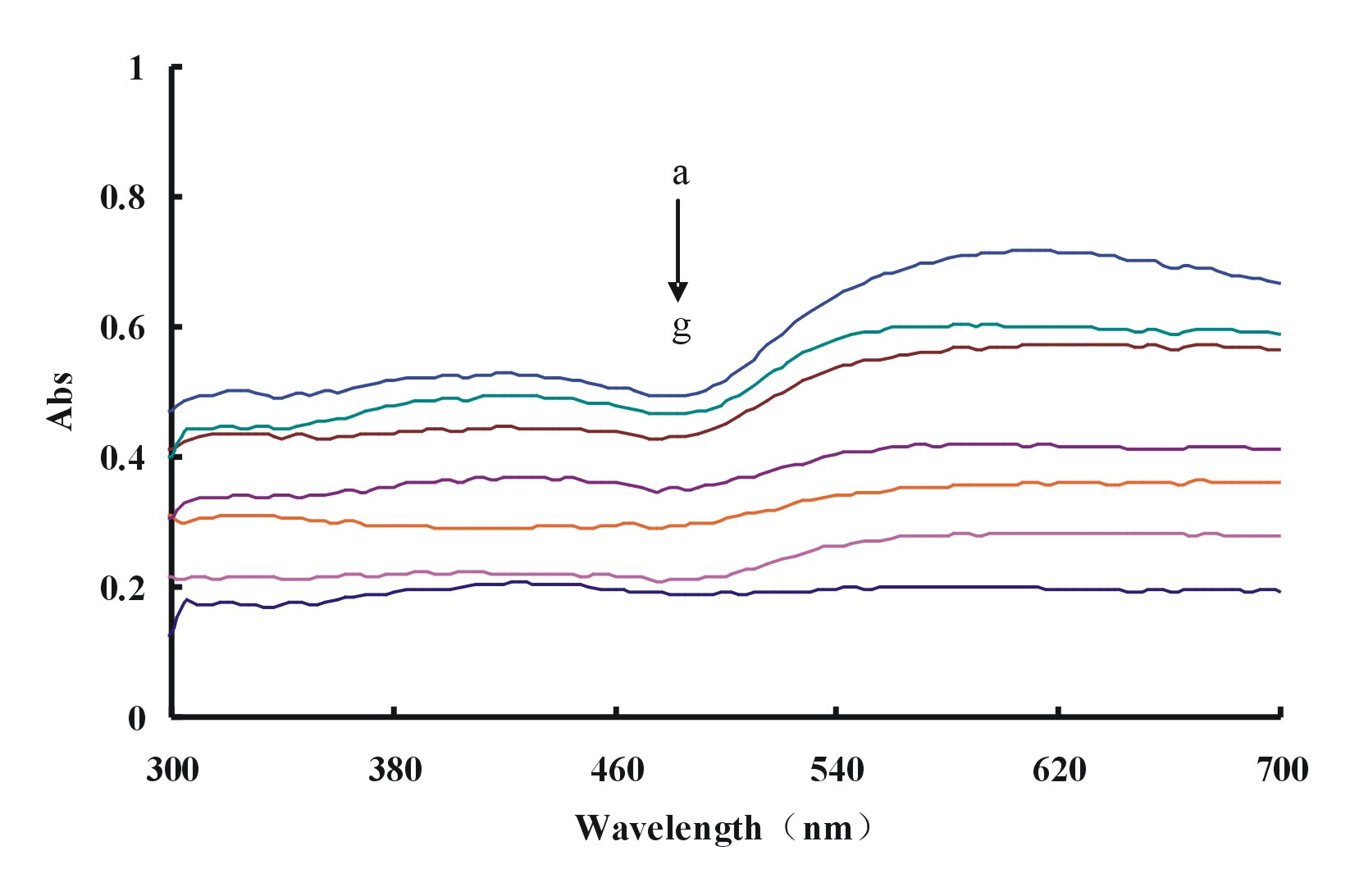


**Fig. S20 Abs spectra of Apt-CD_Ag2_-HAuCl_4_-H_2_O_2_-HCl system**

a: 0.67 mg/mL CD_Ag2_+ 0.16 mmol/L HCl +11.2 μmol/L HAuCl_4_+2 mmol/L H_2_O_2_; b:a+3 nmol/L Apt; c: a+20 nmol/L Apt; d: a+40 nmol/L Apt; e: a+60 nmol/L Apt ; f: a+80 nmol/L Apt; g: a+150 nmol/L Apt


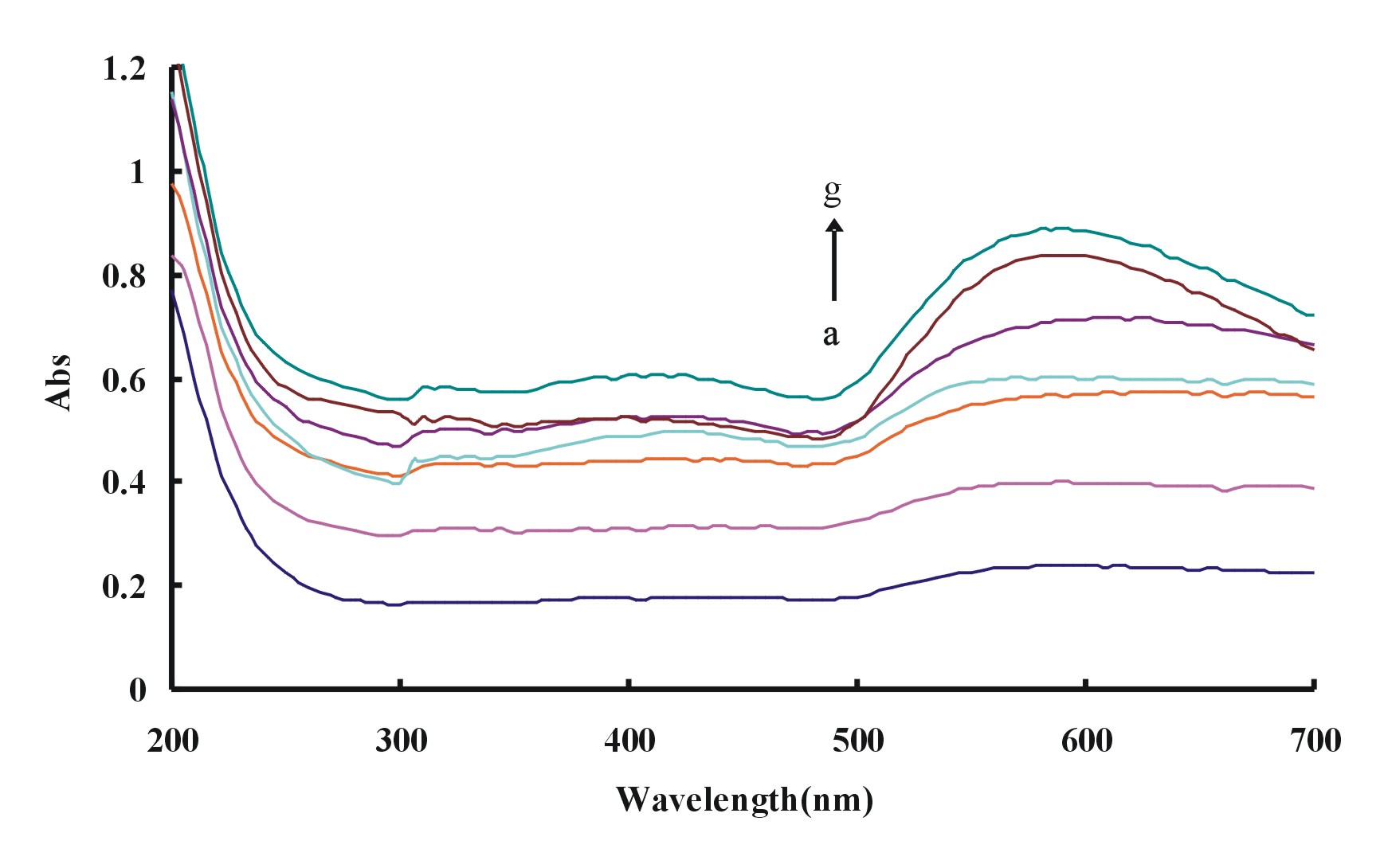


**Fig. S21 Abs spectra of CD_Ag2_-HAuCl_4_-H_2_O_2_-HCl system**

a: 0.16 mmol/L HCl + 11.2 μmol/L HAuCl_4_+2 mmol/L H_2_O_2_; b: a+0.04 mg/mL CD_Ag2_; c: a+0.14 mg/mL CD_Ag2_; d: a+0.25 mg/mL CD_Ag2_; e: a+0.39 mg/mL CD_Ag2_; f: a+0.42 mg/mL CD_Ag2_; g: a+0.59 mg/mL CD_Ag2_.

**
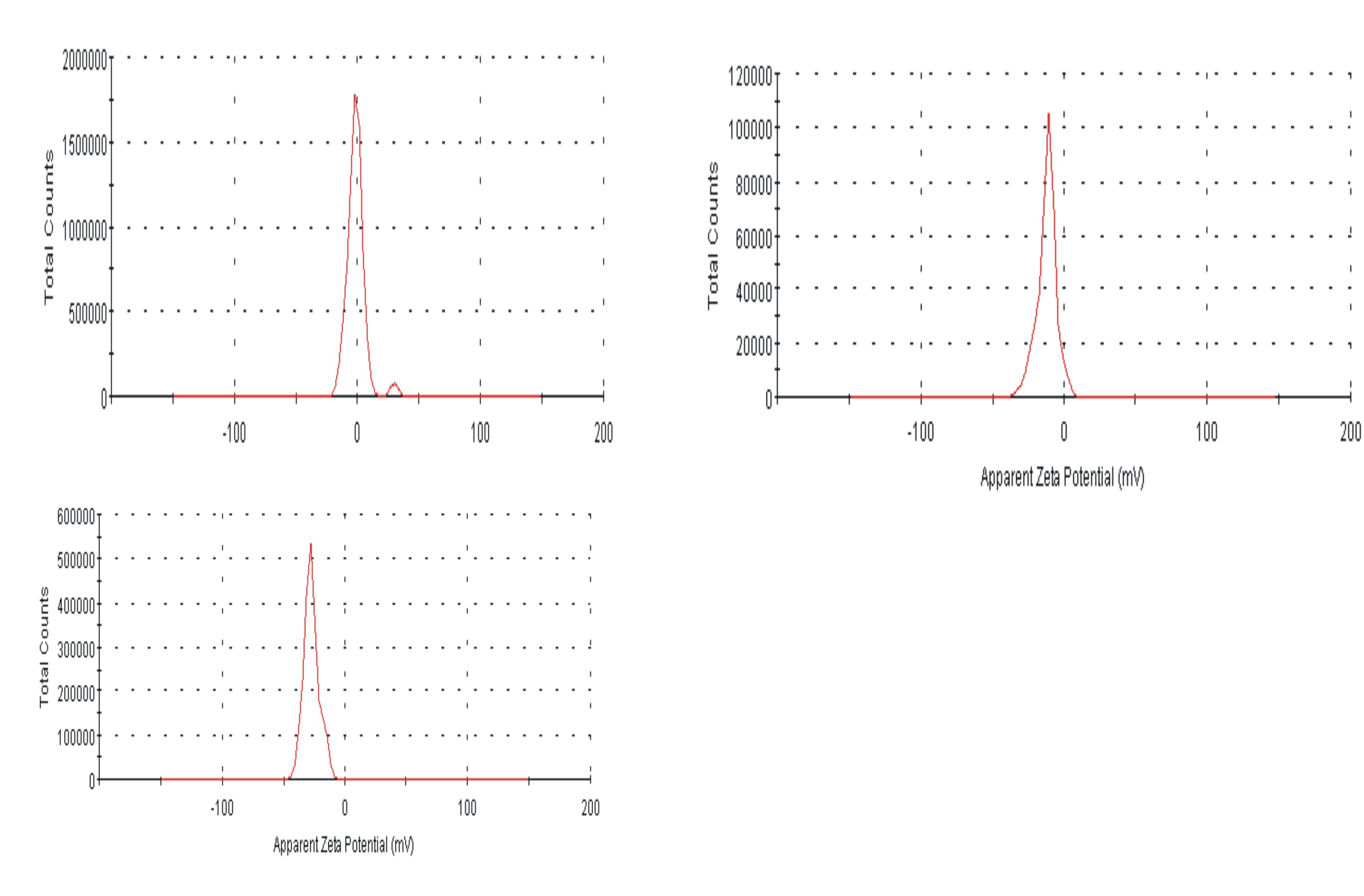
**

-27.2mV

-1.31mV

-12mV

a

b

c

**Fig. S22 Surface charge distribution of Apt-CD_Ag2_-HAuCl_4_-H_2_O_2_-HCl system**

a: 80 nmol/L Apt+ 0.36 mg/mL CD_Ag2_+0.16 mmol/L HCl+11.2 μmol/L HAuCl_4_+2 mmol/L H_2_O_2_; b: a+0.46 μmol/L Pb(II); c:0.36 mg/mL CD_Ag2_.

**
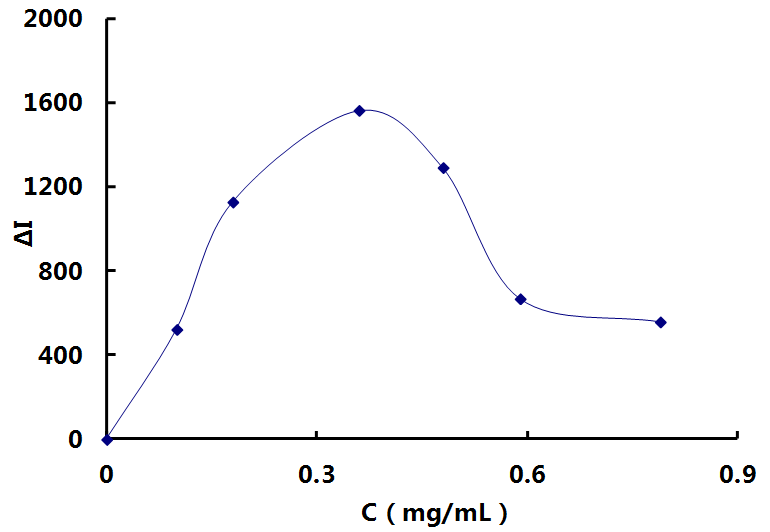
**

**Fig. S23 Effect of the CD_Ag2_ concentration on Δ*I***

10 nmol/mL Apt +0.1 μmol/L Pb(II) + 0.16 mmol/L HCl +11.2 μmol/L HAuCl_4_ +2 mmol/L H_2_O_2_+0.27 μmol/L VBB


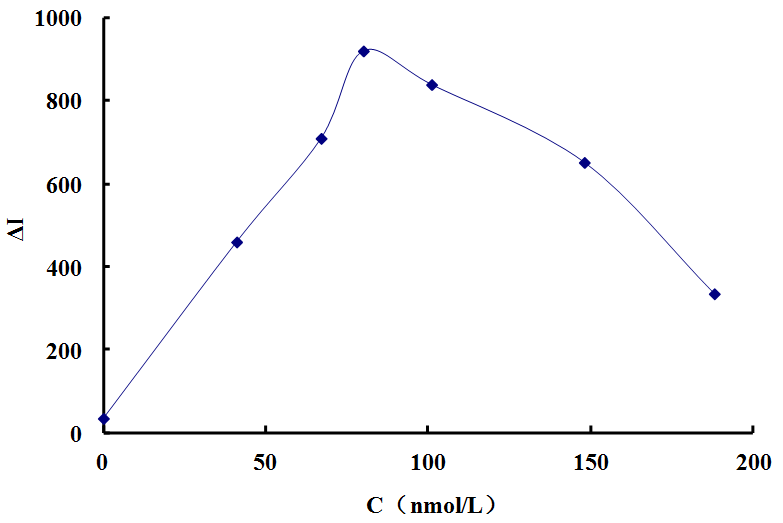


**Fig. S24 Effect of the Apt concentration on Δ*I***

0.1 μmol/L Pb(II)+0.36 mg/mL CD_Ag2_+0.16 mmol/L HCl + 11.2 μmol/L HAuCl_4_ +2 mmol/L H_2_O_2_+0.27 μmol/L VBB


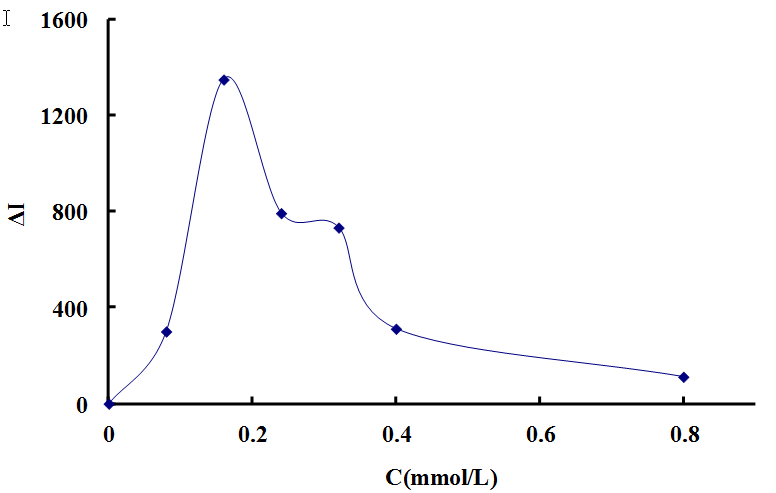


**Fig. S25 Effect of the HCl concentration on Δ*I***

80 nmol/mL Apt +0.1 μmol/L Pb(II)+0.36 mg/mL CD_Ag2_+ 11.2 μmol/L HAuCl_4_ +2 mmol/L H_2_O_2_+0.27 μmol/L VBB


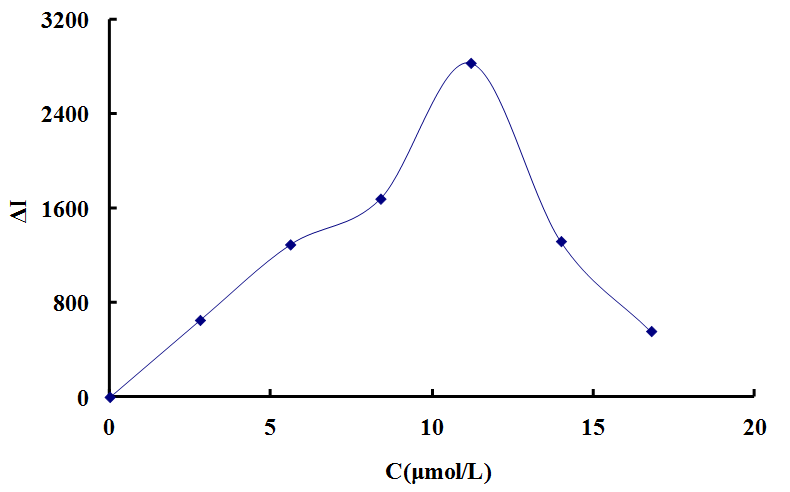


**Fig. S26 Effect of the HAuCl_4_ concentration on Δ*I***

80 nmol/L Apt +0.1 μmol/L Pb(II)+0.16 mmol/L HCl +0.36 mg/mL CD_Ag2_ +2 mmol/L H_2_O_2_+0.27 μmol/L VBB


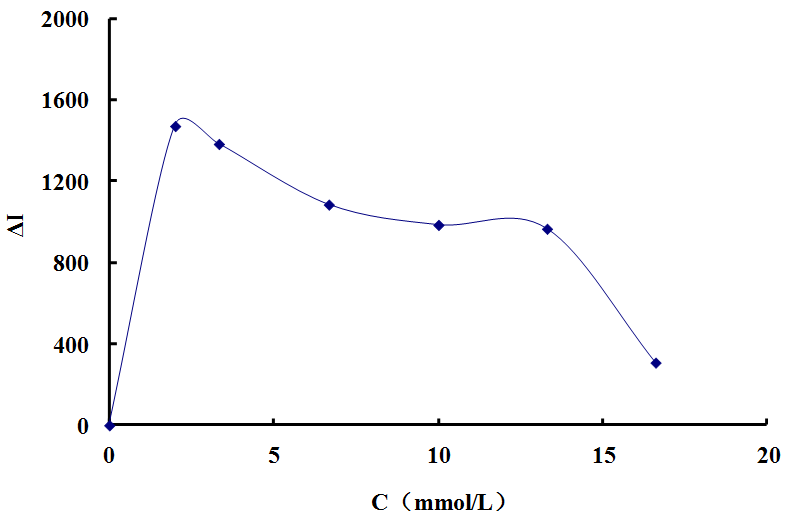


**Fig. S27 Effect of the** H_2_O_2_ **concentration on Δ*I***

80 nmol/L Apt +0.1 μmol/L Pb(II)+0.36 mg/mL CD_Ag2_+0.16 mmol/L HCl +11.2 μmol/L HAuCl_4_+0.27 μmol/L VBB


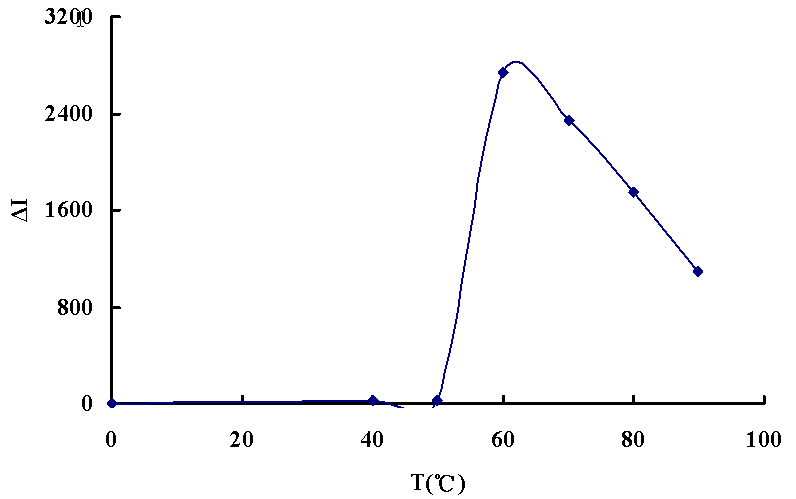


**Fig. S28 Effect of temperature on Δ*I***

80 nmol/L Apt +0.1 μmol/L Pb(II)+0.36 mg/mL CD_Ag2_+0.16 mmol/L HCl +11.2 μmol/L HAuCl_4_+2 mmol/L H_2_O_2_+0.27 μmol/L VBB


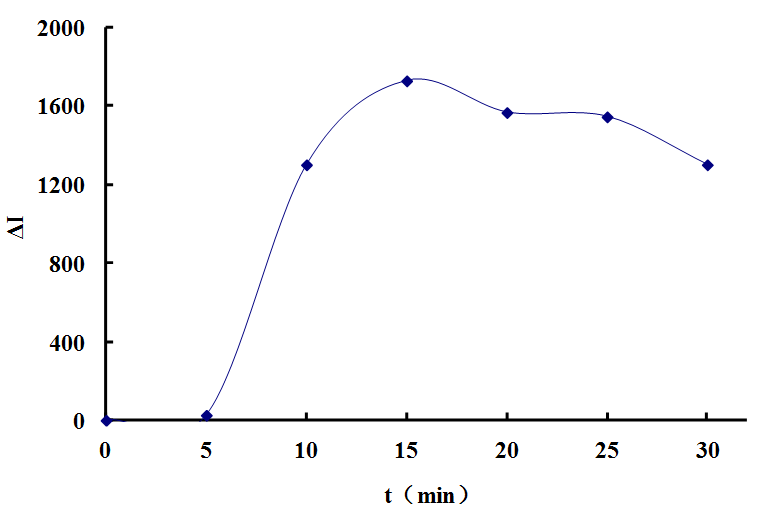


**Fig. S29 Effect of reaction time on Δ*I***

80 nmol/L Apt +0.1 μmol/L Pb(II)+0.36 mg/mL CD_Ag2_+0.16 mmol/L HCl +11.2 μmol/L HAuCl_4_+2 mmol/L H_2_O_2_+0.27 μmol/L VBB

**
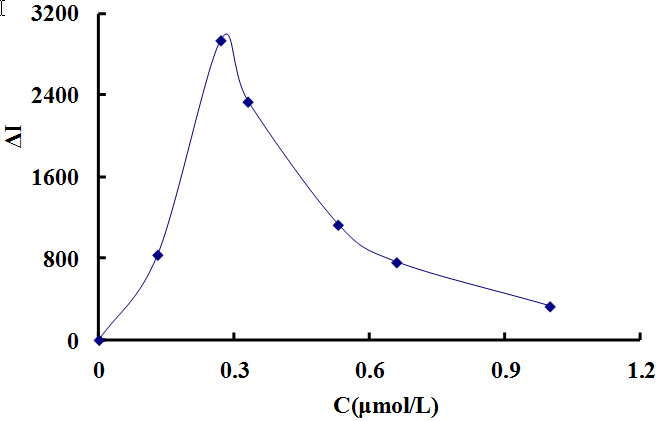
**

**Fig. S30 Effect of the** VBB **concentration on Δ*I***

10 nmol/L Apt +0.1 μmol/L Pb(II)+0.36 mg/mL CD_Ag2_+0.16 mmol/L HCl +11.2 μmol/L HAuCl_4_+2 mmol/L H_2_O_2_

**
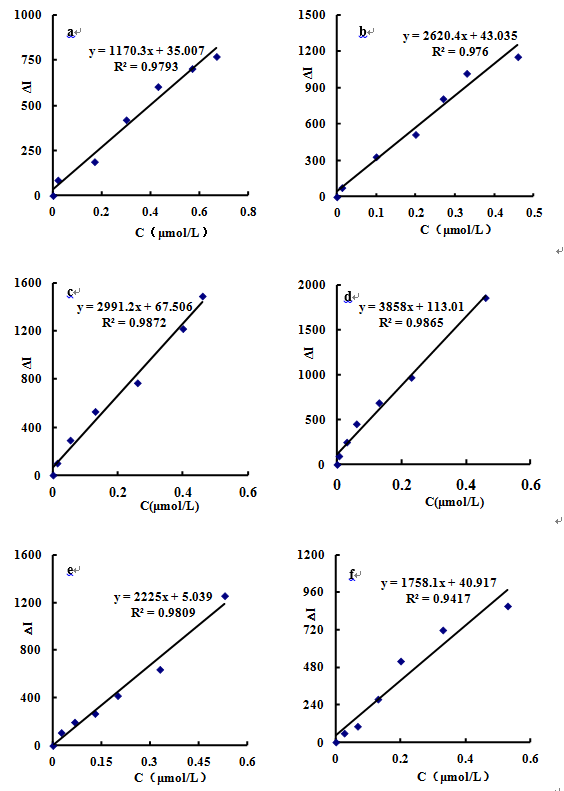
**

**Fig. S31 SERS working curve of Apt-Pb(II)-CD-HAuCl_4_-H_2_O_2_- HCl-VBB system**

a: CD_Ca_ system; b: CD_Ag0_ system; c: CD_Ag1_ system; d: CD_Ag2_ system; e: CD_Ag3_ system; f: CD_Au2_ system.


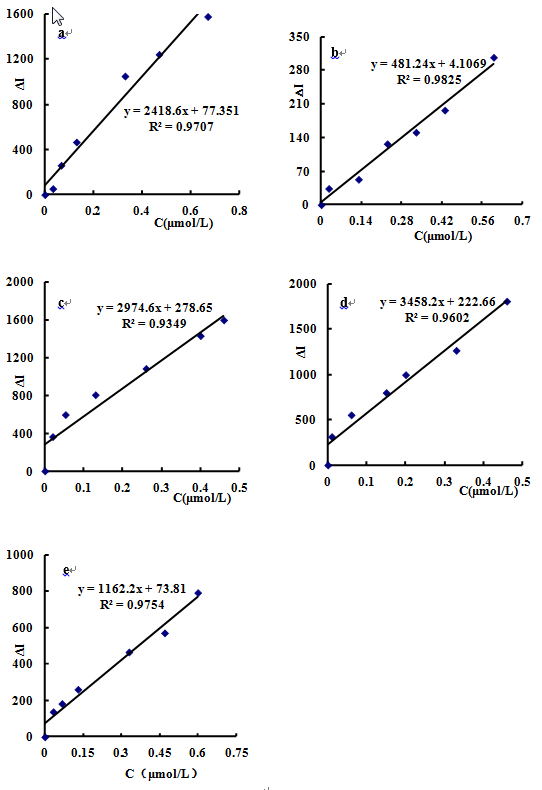


**Fig. S32 RRS working curve of Apt-Pb(II)-CD-HAuCl_4_-H_2_O_2_- HCl system**

a: CD_Ca_ system; b: CD_Ag0_ system; c: CD_Ag1_ system; d: CD_Ag2_ system; e: CD_Ag3_ system.

**
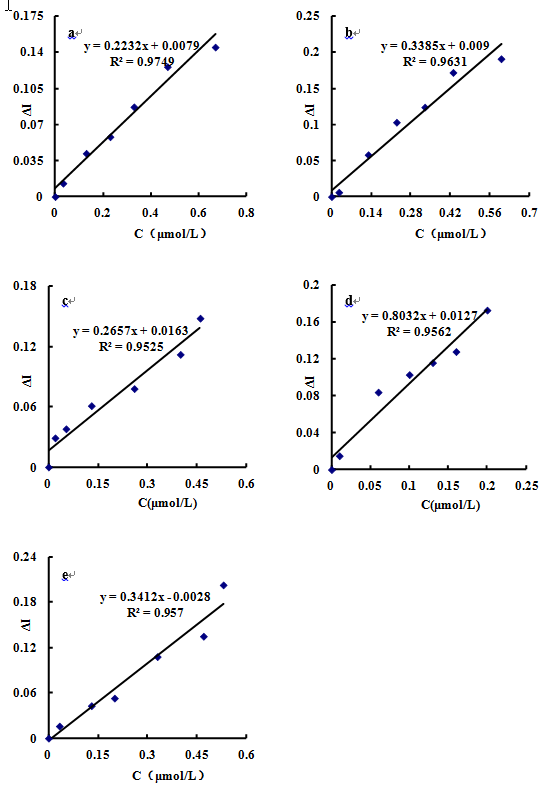
**

**Fig. S33 Abs working curve of Apt-Pb(II)-CD_Ca_-HAuCl_4_-H_2_O_2_- HCl system**

a: CD_Ca_ system; b: CD_Ag0_ system; c: CD_Ag1_ system; d: CD_Ag2_ system; e: CD_Ag3_ system.

Table S1 Comparison of nanocatalytic characteristics

| System | Method | | Linear range | | Regress equation | | Coefficient | |  |
| --- | --- | --- | --- | --- | --- | --- | --- | --- | --- |
| CD_Ca_ | | SERS | | 3.3-30 ng/mL | | ΔI_1615cm-1_=13.943C-16.1 | | 0.9697 | |
|  |  | RRS | | 4-40 ng/mL | | ΔI_375nm_ =11.505C+44.6 | | 0.9382 | |
|  |  | UV | | 4-40 ng/mL | | ΔA_585nm_ =0.0049C+0.01 | | 0.9169 | |
| CD _Ag0_ | | SERS | | 0.06-0.36 mg/mL | | ΔI_1614cm-1_=374.83C-20.6 | | 0.9802 | |
|  |  | RRS | | 0.09-0.96mg/mL | | ΔI_375nm_ =773.82C+44.8 | | 0.9874 | |
|  |  | UV | | 0.09-0.96 mg/mL | | ΔA_575nm_ =0.1948C+0.001 | | 0.9787 | |
| CD _Ag1_ | | SERS | | 0.04-0.36 mg/mL | | ΔI_1614cm-1_=3088C+32.6 | | 0.9818 | |
|  |  | RRS | | 0.04-0.59mg/mL | | ΔI_375nm_ =1246.3C+50.6 | | 0.9516 | |
|  |  | UV | | 0.04-0.59 mg/mL | | ΔA_575nm_ =0.9514C+0.1 | | 0.8869 | |
| CD _Ag2_ | | SERS | | 0.03-0.48 mg/mL | | ΔI_1615cm-1_=4840.9C+196.7 | | 0.9738 | |
|  |  | RRS | | 0.04-0.59 mg/mL | | ΔI_375nm_ =5933.7C+480.9 | | 0.9199 | |
|  |  | UV | | 0.04-0.59 mg/mL | | ΔA_575nm_ =1.0143C+0.1 | | 0.9171 | |
| CD _Ag3_ | | SERS | | 0.04-0.36 mg/mL | | ΔI_1615cm-1_=2284.6C-86.5 | | 0.9387 | |
|  |  | RRS | | 0.04-0.59 mg/mL | | ΔI_375nm_ =4894.8C+290.4 | | 0.9707 | |
|  |  | UV | | 0.04-0.59 mg/mL | | ΔA_575nm_ =0.9356C+0.1 | | 0.9516 | |
| CD _Au0_ | | SERS | | 0.6-6 mg/mL | | ΔI_1619cm-1_=38.649C-10.1 | | 0.9289 | |
| CD _Au1_ | | SERS | | 0.6-6 mg/mL | | ΔI_1619cm-1_=48.834C+13.0 | | 0.9888 | |
| CD _Au2_ | | SERS | | 0.6-6 mg/mL | | ΔI_1619cm-1_=90.552C+11.962 | | 0.9753 | |
| CD _Au3_ | | SERS | | 0.6-6 mg/mL | | ΔI_1619cm-1_=61.325C+8.6212 | | 0.9793 | |
| CD _Au4_ | | SERS | | 0.6-6 mg/mL | | ΔI_1619cm-1_=59.608C+0.6143 | | 0.9943 | |

Table S2 Comparison of aptamer inhibition characteristics

| System | Method | Linear range  nmol/L | Regress equation | Coefficient |
| --- | --- | --- | --- | --- |
| Apt-CD_Ca_ | SERS | 2-102 | ΔI_1615cm-1_=3.2421C+62.0 | 0.9383 |
|  | RRS | 6-150 | ΔI_375nm_ =5.1643C+114.8 | 0.933 |
|  | UV | 4-143 | ΔA_585nm_ =0.0008C+0.02 | 0.9367 |
| Apt- CD_Ag0_ | SERS | 3-92 | ΔI_1614cm-1_=9.001C+18.4 | 0.9709 |
|  | RRS | 4-82 | ΔI_375nm_ =6.4240C+68.6 | 0.9537 |
|  | UV | 4-82 | ΔA_575nm_ =0.0031C+0.05 | 0.9326 |
| Apt- CD_Ag1_ | SERS | 2-80 | ΔI_1614cm-1_=30.442C+135.3 | 0.9677 |
|  | RRS | 4-80 | ΔI_375nm_ =28.678C+58.2 | 0.9539 |
|  | UV | 4-80 | ΔA_575nm_ =0.0063C-0.02 | 0.9599 |
| Apt- CD_Ag2_ | SERS | 1-80 | ΔI_1615cm-1_=88.845C+46.7 | 0.9628 |
|  | RRS | 3-150 | ΔI_375nm_ =34.475C-494.8 | 0.9813 |
|  | UV | 3-150 | ΔA_575nm_ =0.0042C+0.05 | 0.9542 |
| Apt- CD_Ag3_ | SERS | 2-80 | ΔI_1615cm-1_=10.338C+99.5 | 0.9762 |
|  | RRS | 2-103 | ΔI_375nm_ =8.2983C+41.9 | 0.9761 |
|  | UV | 2-60 | ΔA_575nm_ =0.0042C+0.02 | 0.9589 |
| Apt- CD_Au2_ | SERS | 4-103 | ΔI_1615cm-1_=10.338C+99.5 | 0.9762 |

**Table S3 Effect of Interfering Ions on Apt- Pb(II)-CD_Ag2_-HAuCl_4_-H_2_O_2_- HCl-VBB** System

| Coexisting substance | Relative multiple | Relative error (%) | Coexisting substance | Relative multiple | Relative error (%) |
| --- | --- | --- | --- | --- | --- |
| K^+^ | 1000 | 6.4 | Zn^2+^ | 1000 | 9.2 |
| Ca^2+^ | 1000 | 5.3 | Co^2+^ | 100 | -1.4 |
| Na^+^ | 500 | -0.9 | Ba^2+^ | 100 | -3.7 |
| Al^3+^ | 50 | -3.5 | Cl^-^ | 1000 | 6.9 |
| Mg^2+^ | 100 | 1.3 | SO_4_^2-^ | 500 | 4.2 |
| Fe^2+^ | 10 | -8.0 | NO_2_^-^ | 100 | -7.1 |
| NH_4_^+^ | 1000 | 3.9 | S_2_O_3_^2-^ | 500 | 8.1 |
| PO_4_^3-^ | 500 | -4.0 | CO_3_^2-^ | 500 | -2.8 |
| Cu^2+^ | 1000 | 5.1 | Cr^3+^ | 500 | 1.3 |
| Cr^6+^ | 50 | -7.9 | Hg^2+^ | 100 | 6.2 |

**Table S4 Comparation of the reported molecular spectrometric methods of lead**

| Method | Principle of analysis | Linear range  μmol/L | DL | Analysis characteristics | ref. | |
| --- | --- | --- | --- | --- | --- | --- |
| Colorimetric | Pb-Au alloys are formed when the presence of Pb^2+^ ions, which weaken the stability of Au^+^•S_2_O_3_. The 4-mercaptobutanol is enhanced to enter the surface of the AuNP, which lead to its aggregation. The surface plasmon resonance (SPR) absorption of gold nanoparticles is red-shifted and broadened to detect lead ions. | 0.0005-0.01 |  | Complex preparation，  high selectivity high sensitivity. | 41 |  |
| Abs | Double-stranded DNA does not protect AuNP, which aggregates in a high concentration of salt medium, and the color becomes purple. Under the digestion of Pb^2+^, the double-stranded DNA is cleaved into small pieces of DNA to protect the AuNP and its color gradually turns red. | 0.01-0.1 | - | Rapid，high selectivity, low sensitivity. | 42 | |
| Flu | AuNP functionalized graphene was prepared. It has a low fluorescence signal. Pb^2+^ can enhance its fluorescent signal and thus can be used as a method for selectively detecting Pb^2+^. | 0.05-1 | 0.01  μmol/L | High selectivity, complicated operation. | 43 | |
| RRS | Pb^4+^ is reduced to PbH_4_ gas by NaBH_4_, and the gas reacts with Au^3+^ to form nanogold,, which has a resonance Rayleigh scattering signal at 286 nm. A method was established for determining lead ion. | 0.2-33.6 | 0.07 μmol/L | High selectivity, low sensitivity，complicated operation. | 7 | |
| aptamer-SERS | Pb^2+^ reacts with double-stranded DNA to form a small fragment of aptamer, which can stably store AuNP in a high concentration of salt solution. Aptamer-AuNP is used as a nano-simulating enzyme to generate new AuNP particles, and has strong SERS signal after binding to VBB. | 0.017-0.25 |  | high sensitivity, high selectivity, complicated operation. | 44 | |
| SERS | Pb(II) induces a catalytic reaction of DNAzyme, and a SERS sensing method based on deoxyribonuclease is used for detecting lead ion. | - | 0.001  μmol/L | high sensitivity, high selectivity, complicated operation and takes a long time. | 45 | |
| SERS | Aptamer coupled determination of Pb(II) by SERS and by exploiting the reduction of HAuCl_4_ by H_2_O_2_ as catalyzed by Ag-doped Carbon Dots | 0.006-0.46 | 0.0032  μmol/L | Simple, rapid, high sensitivity. | this paper | |
